# Impacts of Extreme Flooding on a Culturally Significant Tree Species: Minnarityi (*Acacia cyperophylla* F. Muell. ex Benth.) in Wangkumarra Country

**DOI:** 10.3390/plants15213356

**Published:** 2026-10-29

**Authors:** Mirritya Ebsworth, Caroline Jones, Rachael V. Gallagher

**Affiliations:** 1 Hawkesbury Institute for the Environment, Western Sydney University, Locked Bag 1797, Penrith, NSW 2751, Australia; m.ebsworth@westernsydney.edu.au; 2 The MARCS Institute for Brain, Behaviour and Development, Western Sydney University, Locked Bag 1797, Penrith, NSW 2751, Australia; caroline.jones@westernsydney.edu.au

**Keywords:** arid zone, climate extremes, culturally significant entities (CSE), extreme events, plant demography, red mulga, seed germination, seed quality, soil chemistry, Traditional Ecological Knowledge (TEK)

## Abstract

Minnarityi (*Acacia cyperophylla*) is a native Australian tree that is of cultural significance to Indigenous Wangkumarra people. Its populations occur along drainage channels, where cyclical flooding likely shapes population dynamics. Climate change-driven changes in hydrological regimes could further alter these dynamics, yet baseline data on populationsin response to floods are lacking from this remote area of inland arid Australia. Here, we investigate the effects of a major flood on minnarityi population dynamics and site conditions across three sites. Pre- and post-flood field surveys assessed population structure, recruitment, mortality, plant condition, and soil characteristics. Subsequent germination experiments using seed collected post-flood explored potential constraints on recruitment. Following the flood, mortality increased and plant condition declined, particularly among seedlings. Despite generally high germination rates in laboratory settings after seed dormancy was broken, evidence of successful recruitment in the field was limited. This suggests that constraints during seedling emergence, establishment, or early survival may limit population recovery. Changes in flood frequency, magnitude, timing, and spatial distribution could shift the balance between mortality and recruitment for this culturally significant species. These findings highlight the need for integrated ecological and cultural approaches to minnarityi conservation and management under changing hydrological regimes.

## 1. Introduction

Floodwaters play a critical role in catchment areas by filling and flushing river systems [1], replenishing wetlands and waterholes [2], and redistributing sediment and organic material across floodplains [2,3]. In inland catchments, these functions are shaped by complex hydrological linkages that connect rivers, floodplains and wetlands across large spatial scales [3], where rainfall often occurs far upstream and takes weeks to move through a system, sequentially connecting otherwise isolated ecological zones [4]. This connectivity is neither consistent nor predictable, and is shaped by the scale, timing and spatial distribution of flows that move across channels, waterholes and floodplains [4,5,6]. In arid and semi-arid dryland river systems, episodic flooding is an important component of natural hydrological variability connecting otherwise isolated aquatic and floodplain habitats and supporting periods of increased ecological productivity [3,4]. These hydrological connections are now being influenced by climate-driven environmental change.

Climate change is increasing global temperatures, altering rainfall patterns and disrupting hydrological cycles, with profound consequences for natural environments [7]. Global mean surface temperatures have recently reached approximately 1.5 °C above pre-industrial levels, contributing to changes in the frequency, duration and intensity of extreme events such as floods, droughts and fires, with significant social, cultural and ecological consequences [8,9]. These changes are also reshaping disturbance regimes that influence plant populations. Among these disturbances, flooding is a key ecological process in riverine and floodplain ecosystems, where timing, magnitude and extent of inundation strongly influences ecosystem structure and function. Changes in rainfall patterns and extreme-event regimes have the potential to reconfigure river channels, accelerate bank erosion and alter habitat structure, with long-term consequences for water quality, biodiversity, and the persistence of long-lived tree species [10,11].

The recruitment and regeneration of tree and shrub species are particularly dependent on episodic flooding [12,13]. In arid and semi-arid environments, where rainfall is low and highly variable, episodic floods provide important pulses of water and resources that support riparian vegetation. Biodiversity in riparian zones in these regions is shaped by the presence of long-lived woody trees and shrubs which function as keystone ecological entities [14,15,16]. These woody species preserve and maintain soil structure, prevent erosion along riparian zones, enhance soil moisture retention, and contribute to water table stability [13,17]. They also provide habitats for fauna, nursery sites for seedlings, and critical shade, nesting sites, food sources and water reservoirs for flora and fauna during periods of prolonged and extreme temperatures [6,13].

Woody vegetation in dryland riparian ecosystems holds profound cultural significance for Indigenous peoples. Traditional Ecological Knowledge (TEK) of these species has developed over millennia, through a direct relationship with the environment, and is embedded within cultural practices, belief systems, language and world views [18,19]. Across the world, First Nations peoples have developed sophisticated knowledge systems and holistic cultural practices through enduring relationships with land, waters, and ecosystems [20,21,22,23].

Australian Aboriginal and Torres Strait Islander peoples have developed TEK that is attuned to the environmental characteristics and variability of their respective Nations [24,25]. Seasonal mobility, sophisticated resource management, and deep ecological understanding, underpinned by kinship relationships [20,26], have enabled communities to live in a reciprocal relationship with Wangkumarra Country, ensuring sustainable use of water, plant and animal resources [27,28,29]. These enduring cultural adaptations exemplify resilience and survival strategies that are both ecologically sound and socially cohesive, reflecting an intimate relationship with Wangkumarra Country [29,30]. The impact of extreme events on culturally significant species must be characterised and ultimately managed so as to maintain and support the retention of TEK [22,31,32,33].

The persistence, recruitment and distribution of culturally significant riparian tree populations is uncertain under climate change. Tree demography is affected by factors such as physical damage, hydraulic uprooting, sedimentation burial, reduced light, and soil anoxia [13,34]. Major flood events have the potential to alter the population demography of large trees, particularly with increases in volumes, flow rates and extended inundation periods [11]. Although projections of future flood regimes remain uncertain, climate models project changes in rainfall patterns and extreme rainfall events across inland Australia [35].

**Figure 1:**
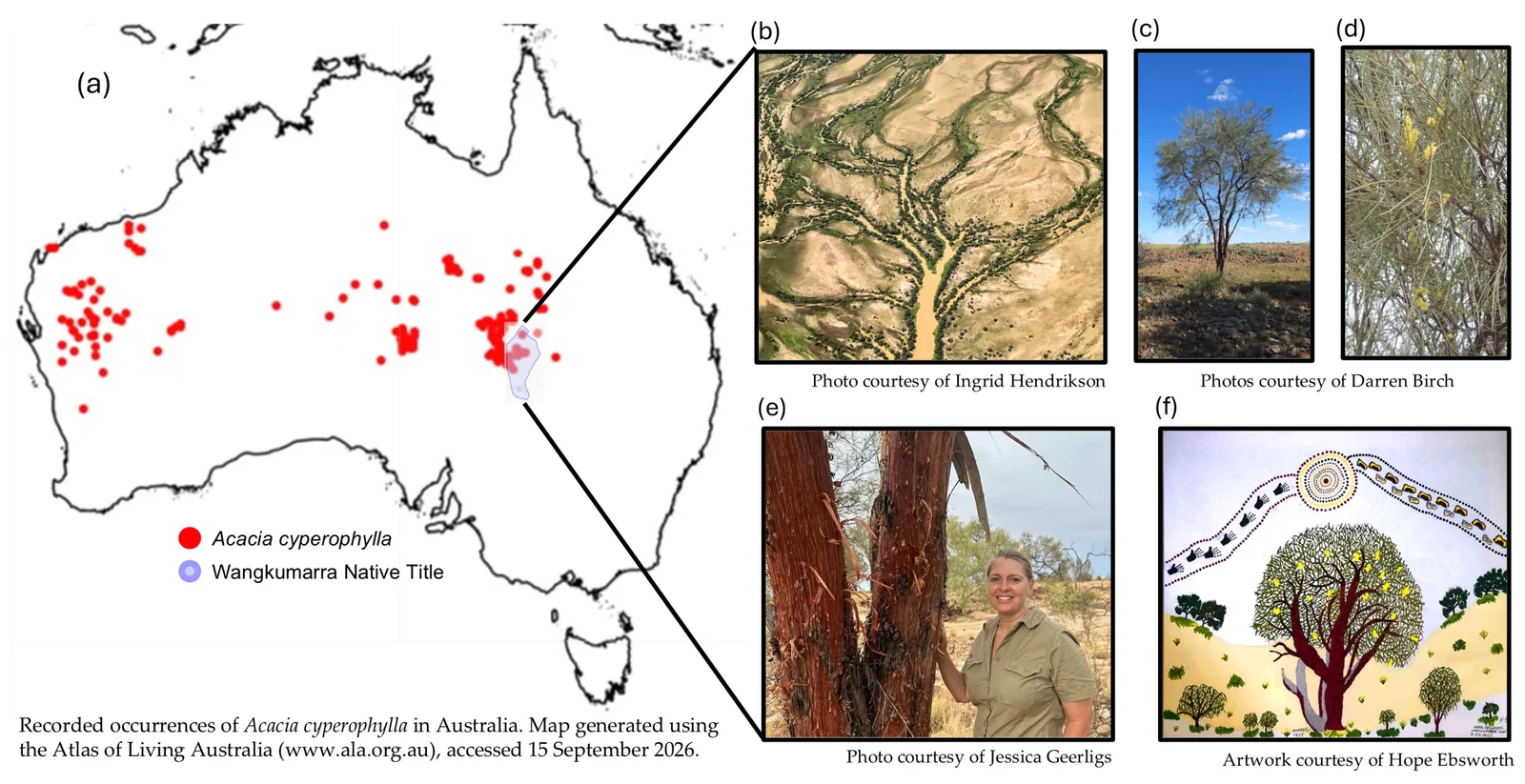
Distribution, morphology, and cultural significance of minnarityi (*Acacia cyperophylla*). (**a**) Minnarityi distribution relative to Wangkumarra Country (purple polygon); (**b**) aerial image of the Channel Country creek system; (**c**) minnarityi tree form; (**d**) minnarityi phyllode and flowers; (**e**) lead author and Wangkumarra woman conducting fieldwork on her country, and (**f**) cultural image of minnarityi, painted (and used with permission) by Wangkumarra Traditional Owner, Hope Ebsworth.

In this study, we examine the effects of extreme flooding on populations of minnarityi (*Acacia cyperophylla*; red mulga or creekline mulga), a culturally significant tree species in Wangkumarra Country (Figure 1). We examine demographic and environmental changes following a major flood event for three minnarityi populations in Wangkumarra Country, including changes in soil conditions, tree condition and survival, and potential regeneration through seed-quality assessment and germination testing. While there is evidence for age–size relationships with flood impacts for other *Acacia* species [36], to date there have been no specific studies of the impact of extreme flooding on the demography, age–size relationships, reproductive and recruitment strategies of culturally significant species, including minnarityi, or the impacts on their population structures as a result of extreme flooding.

To address these knowledge gaps, we assessed the following specific questions:

1. How does extreme flooding influence (a) survival dynamics, (b) population demographic structure, and (c) plant condition of minnarityi (*A. cyperophylla*) in Wangkumarra Country? We expect restructuring of the life-stage profile between pre- and post-flood surveys, with reductions in immature individuals, increases in mortality in mature plants, and a decline in plant condition after flooding.
2. How does extreme flooding alter the environmental conditions that support the establishment and growth of minnarityi? Using data on soil conditions, we quantify chemical changes in composition and relate these to demographic data, anticipating that extreme flooding would alter soil organic matter profiles through sediment deposition and erosion, and that prolonged inundation would redistribute soluble materials that affect soil acidity, salinity, and nutrient dynamics.
3. What is the capacity for regeneration in minnarityi populations in Wangkumarra Country after extreme flooding? We quantify the quality of the post-flood seed cohort and assess the germination capacity to evaluate the availability of putatively viable seeds and the potential for seed-based recruitment in surveyed populations.

Together, these questions provide an integrated assessment of how extreme flooding influences the vulnerability and recovery of a culturally significant arid-zone tree species. By linking changes in population structure, post-flood environmental conditions, and seed-based regeneration, this study advances understanding of how altered and potentially more variable flood regimes associated with climate change may reshape the persistence of culturally important woody species in arid landscapes.

## 2. Materials and Methods

### 2.1. Study Species

Minnarityi was traditionally used by Wangkumarra people for food, medicine, tools, weapons, shelter and ceremonies. Minnarityi has no known central role in Wangkumarra cultural narratives, known as Dreaming Stories, through which knowledge, law, relationships and connections to Wangkumarra Country are conveyed, although the species may hold different cultural significance for other Aboriginal peoples. Although this study focuses on Wangkumarra knowledge and relationships with minnarityi, ethnographic records indicate that *A. cyperophylla* was also culturally important to other Aboriginal peoples of the Channel Country and broader Lake Eyre Basin, including the Mithaka, Kurawali and Maiawali peoples [37]. Minnarityi provides habitat for insects, reptiles, birds and mammals, which in turn provide important food sources for people. The seeds were likely processed for flour, the flowers eaten, and the phyllodes and bark used medicinally. The wood is dense and burns long and hot. The trunks and scaffold branches are relatively straight, ideal for spears and weapons. The trunks were also used for shelters such as *mia mia* (large huts) and grave coverings—the weight of the wood prevented dingoes from digging burial grounds. The bark was used in waterholes to stun fish in sustainable harvesting, akin to similar practices elsewhere, e.g., the Kimberley region of Western Australia [38].

There are 27 *Acacia* species in Australia collectively known by many Aboriginal language groups as minnarityi (the linguistic origins of the term are unclear) [39], and five occur in Queensland: *A. cyperophylla*, *A. chisholmii*, *A. curranii*, *A. lysiphloia*, and *A. monticola*. Of these, *A. cyperophylla* is geographically distinct, distributed along creek lines in arid to semi-arid regions of central Australia. Distribution mapping (Figure 1a) is indicative.

### 2.2. Study Area and Context

Wangkumarra Country refers to the traditional lands and waters of Wangkumarra people, extending across parts of south-western Queensland and north-western New South Wales. The Wangkumarra Native Title determination area (Figure 1a) [40] occurs within parts of the Channel Country bioregion, as defined by the Interim Biogeographic Regionalisation for Australia (IBRA) framework [41,42], so named for its distinct arid-region landscape characterised by extensive river channels, floodplains and associated dryland environments. The landscape is arid to semi-arid and characterised by gibber plains, flood plains, dune fields, clay pans and salt lakes [43]. The area experiences hot summers (average January maximum 30.5 °C, frequent days >40 °C), mild winters (average July minimum 16.8 °C), and low rainfall (sporadic, unpredictable with <200 mm annually) [44]. This highly variable and unpredictable climate has driven the evolution of biota which has adapted to tolerate aridity and episodic inundation [45].

The confluence of the Thompson and Barcoo Rivers forms the Cooper Creek [2], an important inland river [46] within the Lake Eyre Basin, one of the world’s largest internally draining river basins [47]. During major flood events, floodwaters are widely dispersed across the interconnected channels and floodplains of the Cooper system, with flows eventually reaching Kati Thanda–Lake Eyre and, in some events, Lake Blanche via Strzelecki Creek [46]. The 2025 event followed widespread heavy rainfall across the upstream Thomson–Barcoo–Cooper catchment and produced exceptionally high river levels across the system. At the Nappa Merrie gauge, located approximately 5.7–15.0 km from the study sites, the river exceeded 11.03 m, surpassing the gauge recording limit and the 9.0 m threshold for major flooding, as well as the previous documented high of 10.13 m recorded in 1974. Historical records indicate that major floods are a recurrent but irregular and highly variable feature of the Cooper Creek system, both in their timing and magnitude.

### 2.3. Species Surveys

Three sites were surveyed on Nappa Merrie Station in far south-western Queensland (27° 35′ 45″ S, 141° 06′ 22″ E, approximately 80 km ENE of Innamincka, South Australia) during three sampling periods relative to the March 2025 flood event: pre-flood, 3 months and 6 months post-flood. See the Supplementary Materials and Figure S1 for further details. Survey sites were located along Cooper Creek drainage channels adjacent to the Adventure Way and were selected based on the presence of established minnarityi trees and safe site access. At each site, a 50 m × 20 m plot was established perpendicular to the creek channel, extending from the channel midpoint, and GPS coordinates were recorded. The three survey sites were located within the same broader drainage landscape, with Site 1 approximately 15.4 km from Site 2, Site 2 approximately 4.5 km from Site 3, and Site 1 approximately 19.6 km from Site 3. The sites were located approximately 5.7 km, 10.5 km and 15.0 km, respectively, from the Nappa Merrie water gauge. The sites were inaccessible during flooding; therefore, site-specific inundation depth, duration and flow velocity were not measured. Regional hydrological records from the Nappa Merrie, Innamincka and Cullyamurra gauges were used to characterise the magnitude and temporal progression of the broader flood event (Supplementary Figure S1) but were not assumed to represent hydrological conditions at individual sites. The sites are therefore treated as separate sampling locations within the same broader flood-affected landscape rather than independent populations.

The study sites occurred within secondary drainage systems connected to the broader Cooper Creek landscape rather than on the main Cooper Creek channel. Minnarityi occurred as the dominant woody species along these drainage lines, which traversed stony gibber and alluvial landscapes that are characteristic of the south-western Channel Country. Following the 2025 flood, evidence of substantial flow-related disturbance was observed at all sites, including debris lodged within tree canopies, scour around tree bases, and localised channel erosion. However, site-specific flood depth, inundation duration and flow velocity were not measured. Consequently, the relative contributions of prolonged inundation and physical flood disturbance to observed mortality could not be determined. Regional gauge records from the Cooper Creek system provide context for the broader flood event but do not establish the magnitude, duration or source of flow experienced at the study sites. Consequently, it is not possible to determine whether the observed responses resulted predominantly from regional riverine flooding, local runoff within the drainage systems, or a combination of both.

All minnarityi plants—living and dead—within each survey site were measured for plant height (m) and plant condition and assigned a unique identification number (TreeID). Individual plant locations were recorded using the ‘GPS Tracks V5.0.1’ app (DM Software Solutions, Bromley, UK). Plant height was measured using a tape (for smaller plants) and was estimated for larger trees by recording the angle from eye-height of the recorder to the highest point of the canopy using a clinometer.

Plants were assigned to life-stage categories based on height: seedling [1–30 cm tall], sapling [31–100 cm tall], juvenile [>1–3 m tall], mature [>3 m tall]. These height thresholds were developed for the present study to provide a consistent classification of demographic structure and have not been formally validated for *A. cyperophylla*. While height may not correspond directly to plant age in long-lived arid-zone woody species, it provided a practical and repeatable basis for classifying individuals during field surveys. Plant condition was assessed visually based on the proportion of canopy showing damage or decline and classified as: good [<33% canopy damage or decline], fair [34–66% canopy damage or decline], poor [>67% canopy damage or decline], dead-standing [trunks with upright snags; no visual evidence of living material; decay or decomposition evident], dead-fallen [trunks horizontal; remnant trunks without snags; no visual evidence of living material; decay or decomposition evident]. Dead-standing and dead-fallen individuals were subsequently combined into a single ‘Dead’ category for statistical analysis.

### 2.4. Soil Sampling

Samples were collected using an auger and pooling samples from 5 cm deep cores from three locations along the midline of each site, after removing above-ground plant material and detritus. Newly deposited flood sediment was not sampled separately from the underlying soil; therefore, differences in chemical properties between flood-deposited sediment and pre-existing soil could not be assessed. Soil was sent for analysis testing at the Environmental Analysis Laboratory (Southern Cross University) for physical qualities, micro-nutrient and macro-nutrient profiles.

### 2.5. Seed Quality Testing

Fresh seed was collected from three randomly selected trees at each site in November 2025, eight months post-flood. As no pre-flood seed cohort was available for comparison, the post-flood seed assessment was intended to characterise seed quality and germination capacity at the time of collection, rather than to test whether flooding affected seed quality. Mature pods were collected from the ground, except for one tree (Tree 3, Site 3, pods from tree). Seeds were stored separately in labelled paper bags and maintained at ambient temperature until seed assessment and germination trials were conducted.

Seed content (quality) was assessed using X-ray imaging with a Kubtec Scientific Paramater^®^ Cabinet X-ray System (Stratford, CT, USA) at PlantBank, at the Australian Botanic Garden, Mount Annan, NSW. Seeds from each tree were randomly arranged in either (4 × 4) or (5 × 5) grids on imaging trays, depending on seed size, to prevent overlap and enable identification of individual seeds. Each tray was X-rayed to visualise embryo development and internal seed structure with instrument settings subsequently adjusted to capture a corresponding surface image, both at 2× and 3× magnification.

Seed quality was determined by visual inspection of the X-ray images. Seeds were classified as putatively viable and included in the subsequent germination trial when X-ray images indicated a fully developed, intact embryo and endosperm with no visible evidence of internal damage [48,49,50]. Seeds not meeting these criteria were excluded from the subsequent germination trial. These included seeds that were empty (lacking internal tissues), and had poorly developed embryos and/or extensive insect damage (e.g., bore holes or tissue damage) [51].

The total mass of seeds from each tree was measured using a Mettler AE260 Delta analytical balance (Mettler Instrumente AG, Greifensee, Switzerland) to the nearest 0.0001 g, after subtracting the mass of the storage envelope. Seed mass was then standardised and expressed as grams per 1000 seeds (i.e., g 1000 seeds^−1^).

### 2.6. Seed Germination Testing

Following X-ray assessment, suitable seeds were randomly assigned to one of three pre-germination treatments: control (Ctl—no pre-treatment), hot water (HW) and mechanical scarification (Sc). Allocation among Site × Treatment combinations was unequal because the number of suitable seeds available varied among collection sites. The numbers of seeds and Petri dishes allocated to each Site × Treatment combination are provided in Supplementary Table S3. Seed heteromorphism was not specifically assessed in this study. However, variation among individual seeds in dormancy and imbibition responses has been reported across Australian *Acacia* species and may contribute to within-population variation in germination responses [52].

In the hot water treatment, approximately 250 mL of water was brought to the boil before seeds were immersed for 2 min (immersion temperature ~93 °C and removal temperature ~86 °C).

In the scarification treatment, the seed coat was mechanically nicked along the seed margin using sanitised nail clippers, taking care not to damage the embryo. In all conditions, seeds were transferred to Petri dishes using sanitised forceps, in fixed, equidistant positions on three sheets of single ply paper towel moistened with pre-sterilised (boiled) water in a 90 mm Petri dish. Two additional sheets of moistened paper towel were placed over the seeds before the lid was fitted. Each seed was assigned a unique identifying number (1–6).

In total, 60 Petri dishes were prepared and arranged on a (6 × 10) grid. Initial dish positions were randomised and re-randomised every second day. All dishes were exposed to natural diurnal fluctuations and ambient temperatures (mean range 15.6–20.7 °C).

Petri dishes were inspected daily for the first 28 days and every second day thereafter until day 40. A seed was considered germinated when the radicle had emerged to a length of at least 2 mm [53,54]. Germinated seeds were removed from dishes immediately after assessment. Seeds exhibiting fungal contamination were also immediately removed and paper towel replaced in affected dishes, with all remaining seeds returned to their original numbered positions. As a preventative hygiene measure, the paper towel in all Petri dishes was replaced weekly, and forceps were sanitised between handling individual seeds. After 40 days, the percentage of seeds germinated in each dish was calculated (% germination).

### 2.7. Statistical Analyses

All statistical analyses and figures were generated in R version 4.5.2 [55] using the following packages: dplyr [56], emmeans [57], ggplot2 [58], ggrepel [59], lme4 [60], lmtest [61], nlme [62], ordinal [63], readxl [64], tidyr [65], and vegan [66]. All statistical tests were considered significant at a *p*-value of 0.05. Microsoft Co-Pilot was used to assist with data cleaning, statistical code generation, and manuscript editing. The authors reviewed and edited the code and content, taking full responsibility for all content.

#### 2.7.1. Survival 

Baseline population structure was established from the raw counts of all individuals (living or dead) recorded pre-flood, while survival and transition analyses involved only living individuals.

To investigate survival dynamics after the flood (Question 1a), plants recorded as alive at the pre-flood survey were re-assessed at both post-flood surveys using their TreeID and were classified as being either alive (1) or dead (0). Plants that could not be located at their recorded positions during post-flood surveys were classified as missing/lost. For the primary survival analysis, both visibly dead and missing/lost individuals were assigned a survival status of 0, while living individuals were assigned a status of 1. Because the fate of missing individuals could not be confirmed, a sensitivity analysis was conducted by repeating the Post6 survival models after excluding all missing/lost individuals. A logistic regression was fitted using a binomial error distribution and logit link function, using pre-flood plant heights as a continuous predictor variable.

Changes in population demographic structure (Question 1b) were assessed by comparing the abundance (count) of individuals living in each life stage across the pre-flood and post-flood surveys.

Survival differences were assessed using a binomial generalised linear model (GLM) with a logit link function [55], using pre-flood life stage (seedling, sapling, juvenile, mature) as a predictor. Model significance was assessed using likelihood ratio chi-square tests, and pairwise comparisons among life stages were conducted using estimated marginal means where significance was detected.

Recruits were defined as seedlings absent from the pre-flood survey (Pre) and first recorded during a post-flood survey (Post3 or Post6). The recruitment rate was calculated as follows:

(1) $$R\left(\%\right)=\left(\frac{N_{r}}{N_{0}}\right)\times 100$$

where

*N_r_* = number of recruits detected after flooding;

*N*_0_ = number of living individuals recorded during the pre-flood survey.

The dead-or-lost rate was calculated as the percentage of pre-flood living individuals that were either visibly dead or missing/lost at the Post6 survey, using the following formula:

(2) $$M\left(\%\right)=\left(\frac{N_{d}}{N_{0}}\right)\times 100$$

where

*N_d_* = number of individuals recorded as visibly dead or missing/lost by the final survey;

*N*_0_ = number of living individuals recorded during the pre-flood survey.

Plant condition was assessed at each survey and classified as either good, fair, poor, or dead. Condition composition was summarised as the number and proportion of individuals within each condition category.

Temporal changes in plant condition were analysed using cumulative link mixed models (CLMM) with a logit link function. Condition score was treated as an ordinal response variable, survey period (Pre, Post3, Post6) as a fixed effect, and individual tree identity (TreeID) was included as a random effect to account for repeated measures.

The significance of survey period on plant condition (Question 1c) was assessed using likelihood-ratio tests comparing models with and without the survey period effect. Where significant effects were detected, estimated marginal means were calculated and pairwise comparisons among survey periods were conducted using Tukey-adjusted tests to identify differences between surveys.

To determine whether pre-flood plant condition influenced subsequent survival, the distribution of pre-flood condition scores was compared between individuals that survived and those that died by the end of the monitoring period. Because condition scores were ordinal, differences between survival groups were evaluated using a Wilcoxon rank-sum test.

#### 2.7.2. Soil Chemistry Analysis

Soil chemical variables represented five broad aspects of soil function: salinity and acidity (electrical conductivity [EC; dS m^−1^], exchangeable sodium [Na^+^; cmol^+^ kg^−1^], pH); nitrogen dynamics (nitrate [NO_3_-N; mg kg^−1^], ammonium [NH_4_-N; mg kg^−1^], total nitrogen [Total N; %]); Organic Matter (total carbon [Total C; %]); nutrient availability (extractable sulfur [S; mg kg^−1^], phosphorus [P; mg kg^−1^]); and soil fertility (exchangeable potassium [K^+^; cmol^+^ kg^−1^], exchangeable calcium [Ca^2+^; cmol^+^ kg^−1^], exchangeable magnesium [Mg^2+^, cmol^+^ kg^−1^], effective cation exchange capacity [ECEC; cmol^+^ kg^−1^]).

Values reported below analytical detection limits were assigned one-half of the reported detection limit prior to analysis, following standard practice for environmental datasets containing small proportions of left-censored observations [67].

Differences in soil chemistry among survey periods (Question 2) were explored using principal component analysis (PCA) and permutational multivariate analysis of variance (PERMANOVA). Variables were standardised to a mean of zero and standard deviation of one prior to analysis to account for differences in measurement units and scales. PCA was performed using the prcomp() function and the first two principal components were used to visualise multivariate patterns in soil chemistry among survey periods. PERMANOVA was performed using the adonis2() function in the vegan package [66], based on Euclidean distances calculated from the standardised soil chemistry data. Survey period was included as a fixed factor, and statistical significance was assessed using 999 permutations.

To facilitate comparison among individual soil variables, values were additionally standardised as *z*-scores within each variable. Standardised values were visualised using a heat map, with positive values indicating measurements above the variable mean and negative values indicating measurements below the variable mean. This approach was used to assess the relative direction and magnitude of temporal changes among soil properties measured on different scales.

Individual soil variables were summarised descriptively for each survey period using means and measures of variability. Percentage changes relative to pre-flood conditions were additionally calculated to describe the direction and magnitude of observed differences. These individual-variable changes were not subjected to separate inferential tests and are therefore interpreted as descriptive patterns only.

#### 2.7.3. Seed Analysis

X-ray-assessed seed quality was summarised as the proportion of seeds classified as putatively viable or discarded relative to the total number of seeds collected at each site. Germination success was calculated as the proportion of seeds that germinated during the germination experiment.

Germination was treated as a binary variable, with seeds scored as either germinated (1) or not germinated (0). Mean time to germination (MTG, days) was calculated for each site as the average time required for seeds to germinate, weighted by the number of seeds germinating on each observation day, using the following formula:

(3) $$\mathrm{MTG}=\frac{Ʃ(n_{i}t_{i})}{Ʃn_{i}}$$

where

*n_i_* = number of seeds germinating on day *t_i_*.

*t_i_* = time (days) from the start of the experiment to germination.

Ʃ*n_i_* = total number of germinated seeds.

Cumulative germination percentages were calculated through time for each treatment and plotted to compare germination rates and final germination success among treatments.

The effects of seed origin (Sites 1–3) and dormancy-breaking treatment (control, hot water, scarification) on germination success were evaluated using a generalised linear mixed model (GLMM) with a binomial error distribution and logit link function [55]. Site and treatment were included as fixed effects, while plate identity was included as a random effect to account for the non-independence of seeds within plates. A Site × Treatment interaction was also evaluated; however, sparse replication within some Site × Treatment combinations, including combinations represented by only one or two Petri dishes, resulted in unstable interaction estimates. The interaction was therefore not retained in the final model. Germination models were fitted using maximum likelihood estimation [60]. Odds ratios, regression coefficients, standard errors, *z*-statistics and *p*-values were used to assess the influence of site and treatment on germination probability.

## 3. Results

### 3.1. Survival Dynamics

A total of 252 minnarityi plants were recorded across the three sites before flooding, comprising individuals across the seedling, sapling, juvenile and mature life-stage categories. Of these, 72 were already dead in the pre-flood survey and were excluded from subsequent analyses. Of the 180 living pre-flood individuals, 116 remained alive at Post6, 45 were visibly dead and 19 were missing/lost. The total number of living plants at the surveyed sites was 125 at Post6, comprising 116 surviving pre-flood individuals and nine post-flood recruits.

**Figure 2:**
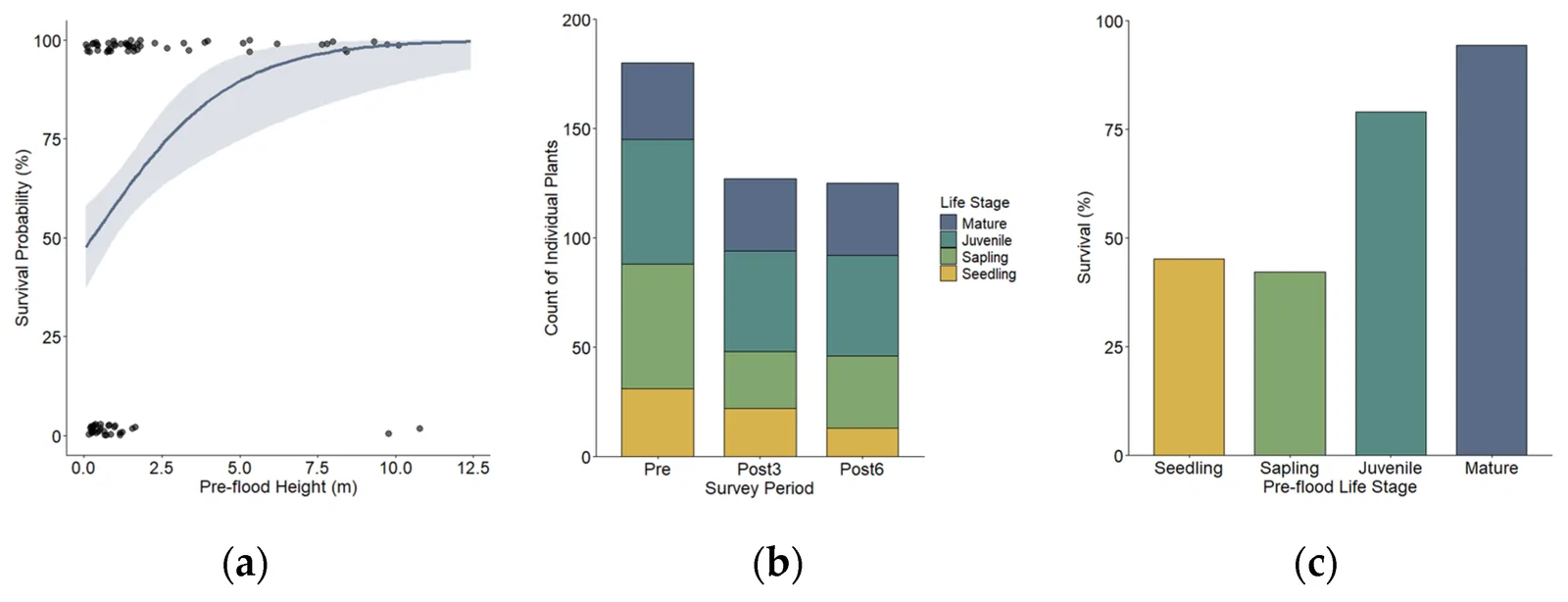
Population demographic change after extreme flooding of minnarityi (*Acacia cyperophylla*) in Wangkumarra Country. (**a**) Relationship between pre-flood plant height and post-flood survival. Points represent individual plants (1 = survived, 0 = dead/lost), the solid line shows the fitted logistic regression, and the shaded area represents the 95% confidence interval. (**b**) Abundance of living individuals within each life stage before flooding (Pre), three months after flooding (Post3), and six months after flooding (Post6). Colours indicate life stages, and stacked columns represent the total number of living individuals per survey period. (**c**) Survival (%) of pre-flood individuals six months after flooding, by life stage.

Logistic regression revealed a significant positive relationship between pre-flood plant height and survival probability when visibly dead and missing/lost individuals were combined (β = 0.456 ± 0.135, *z* = 3.38, *p* < 0.001; Figure 2a), with larger individuals being more likely to survive. This relationship remained significant when the 19 missing/lost individuals were excluded in the sensitivity analysis (β = 0.324 ± 0.116, *z* = 2.80, *p* = 0.005).

### 3.2. Population Demographic Structure

Extreme flooding affected life stages differently (Figure 2b). Seedling counts decreased by 58% between the pre-flood and Post6 surveys, while sapling and juvenile counts decreased by 42% and 19%, respectively. In contrast, mature trees showed little change in abundance, declining by only 6%. Correspondingly, survival 6 months post-flood varied significantly among life stages (binomial GLM, likelihood-ratio test: χ^2^ = 40.02, df = 3, *p* < 0.001; Figure 2c; Table 1). This effect remained significant when missing/lost individuals were excluded in the sensitivity analysis (χ^2^ = 21.99, df = 3, *p* < 0.001). Mature trees showed the highest confirmed survival (94%), followed by juveniles (79%). When missing/lost individuals were classified as non-survivors, seedling and sapling survival were 45% and 42%, respectively. When missing/lost individuals were excluded, estimated survival increased to 58% for seedlings and 53% for saplings, without altering the overall conclusion that survival differed among life stages. Relative to mature trees, the odds of survival were significantly lower for saplings and seedlings, and marginally lower for juveniles (Table 1). Juveniles survived significantly better than seedlings and saplings, while no difference in survival was detected between seedlings and saplings (Table 1).

After flooding, individuals either remained in the same life stage (stable), progressed to a more advanced life stage (growth), or were lost through mortality or flood-related displacement (dead/lost) (Supplementary Table S4). Of the 180 plants alive before the flood, 116 remained alive at the final survey, 45 were visibly dead and 19 were missing/lost. The resulting confirmed mortality rate was 25.0%, while the combined dead-or-lost rate was 35.6% over the six-month survey period.

Nine recruits were detected during the 3-month post-flood survey (*R* = 0.05), comprising eight individuals at Site 2 and one individual at Site 3. Recruits were identified based on their absence from the pre-flood survey and subsequent appearance during post-flood surveys. The origin of these individuals was not investigated, and it was therefore not possible to determine whether all recruits originated from seed or whether some arose through vegetative regeneration. No new recruits were recorded at any sites during the Post6 survey. Detailed life-stage transition data are provided in Supplementary Table S4 and are presented as a demographic baseline for future monitoring.

### 3.3. Plant Condition

**Figure 3:**
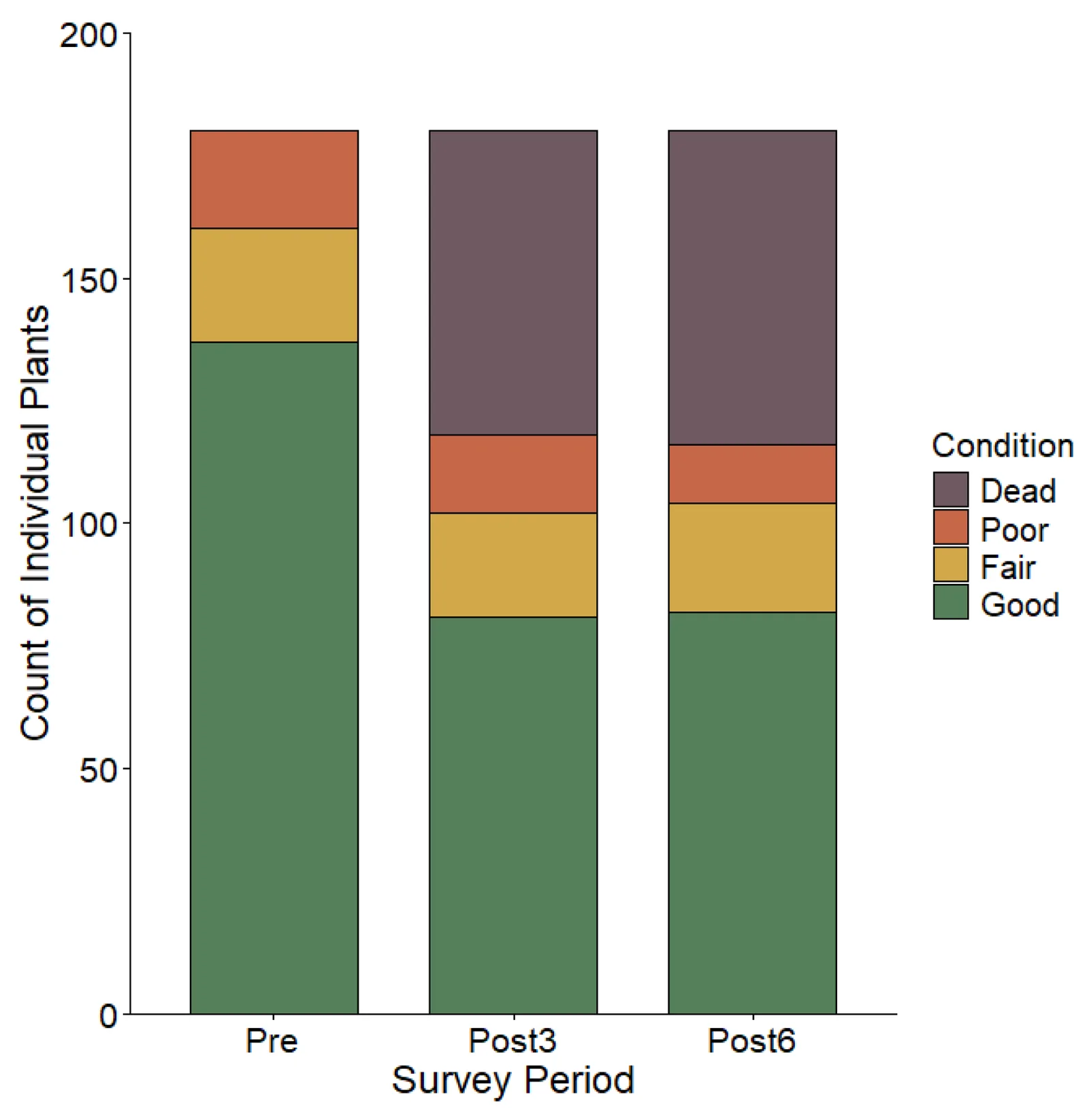
Condition of individual minnarityi (*Acacia cyperophylla*) plants before flooding (Pre), three months after flooding (Post3), and six months after flooding (Post6). Stacked bars represent counts of individual plants classified as good, fair, poor, or dead during the pre-flood survey (Pre), three-month post-flood (Post3), and six-month post-flood (Post6) surveys.

Prior to flooding, most minnarityi plants were in good condition (137 individuals; 76%), with fewer in fair (23 individuals; 13%) or poor (20 individuals; 11%) condition. Plant condition declined significantly following flooding (CLMM likelihood ratio test: χ^2^_2_ = 123.9, *p* < 0.001), with condition remaining similarly poor at both post-flood surveys (Post3 vs. Post6: *p* = 0.96). The number of minnarityi plants in good, fair and poor condition declined by 40%, 4% and 42%, respectively, accompanied by a marked increase in mortality, with 34% and 36% of individuals classified as dead at the Post3 and Post6 surveys, respectively (Figure 3).

Pre-flood condition was not associated with mortality, as individuals classified as being in poor condition were no more likely to die than those classified as fair or good (Wilcoxon rank-sum test: W = 4100.5, *p* = 0.120).

Of the nine recruits recorded at the Post3 survey, seven were in good condition and two in poor condition. All recruits remained in the same condition in the Post6 survey.

### 3.4. Soil Chemistry Composition

**Figure 4:**
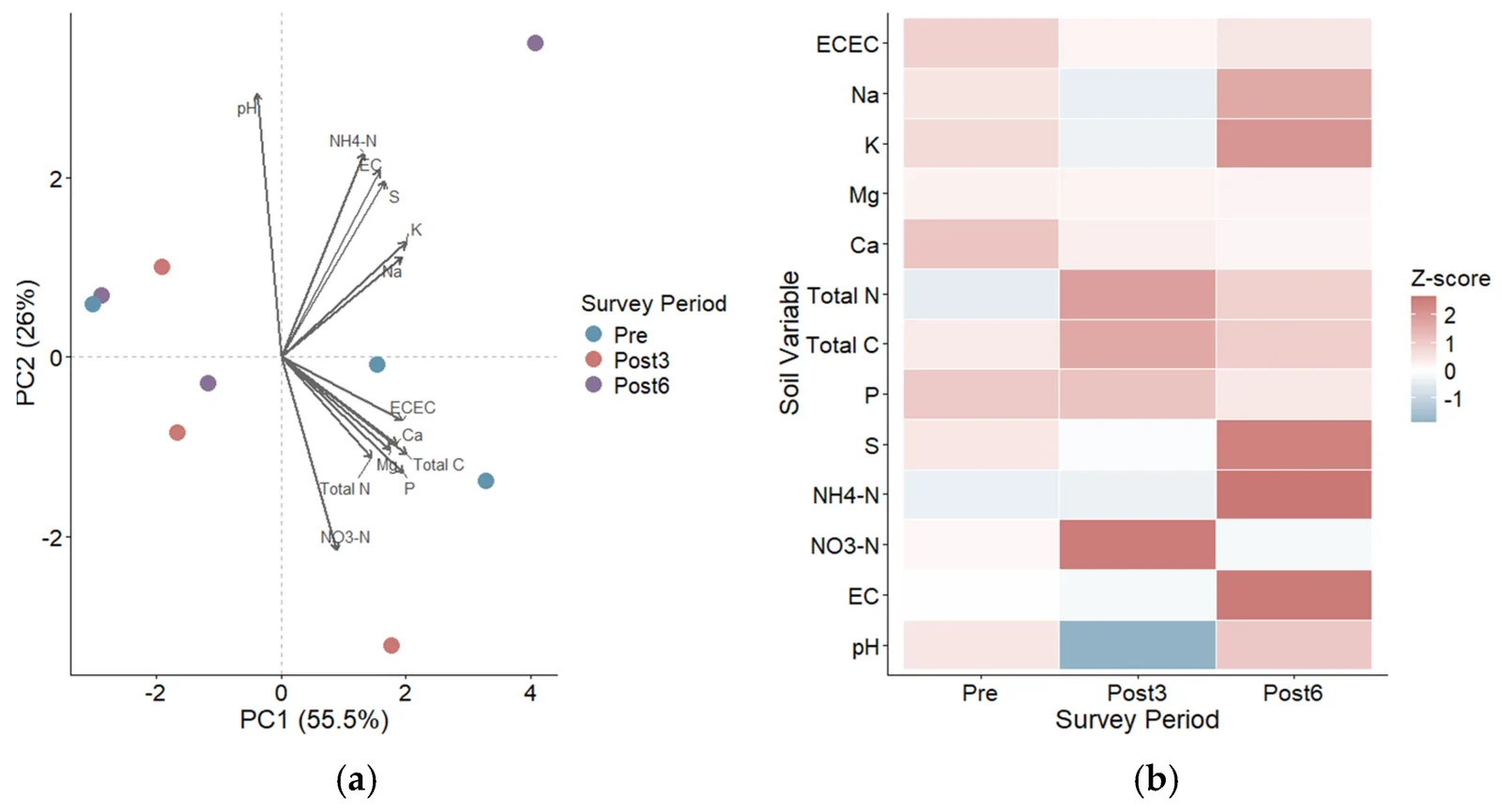
Soil chemistry before and after extreme flooding at sites occupied by minnarityi (*Acacia cyperophylla*) in Wangkumarra Country. (**a**) Principal component analysis (PCA) biplot of soil chemical properties measured before flooding (blue circles), three months after flooding (red circles), and six months after flooding (purple circles). Points represent soil samples coloured by survey period, and arrows indicate the direction and strength of individual soil variables. (**b**) Heat map of standardised (*z*-score) soil chemical properties across survey periods. Values were standardised within each soil variable to facilitate comparison among variables measured on different scales. Red indicates values above the variable mean, blue indicates values below the mean, and white indicates values near the mean.

Overall, soil chemistry showed limited responses to flooding. Principal component analysis (PCA) indicated substantial overlap among pre-flood, Post3 and Post6 samples (Figure 4a), with the first two principal components explaining 81.5% of the total variation. See Supplementary Materials and Table S1 for further details. Consistent with the PCA, PERMANOVA detected no significant differences in overall soil chemistry among survey periods (F_2,6_ = 0.684, R^2^ = 0.186, *p* = 0.644). Mean soil pH was 7.75 ± 0.38 before flooding, 7.34 ± 0.91 at Post3, and 8.07 ± 0.43 at Post6 (Supplementary Table S2).

Although no significant difference in overall soil chemistry was detected among survey periods, descriptive patterns were evident for some individual soil variables (Figure 4b). Following flooding, higher concentrations of NH_4_-N, EC, S and total N were observed, while Ca^2+^, ECEC, Mg^2+^, P and total C were generally lower. These individual patterns were not tested independently and should therefore not be interpreted as statistically demonstrated changes. Underlying values and variability among sites are provided in Supplementary Table S2.

### 3.5. Seed Quality and Germination

**Figure 5:**
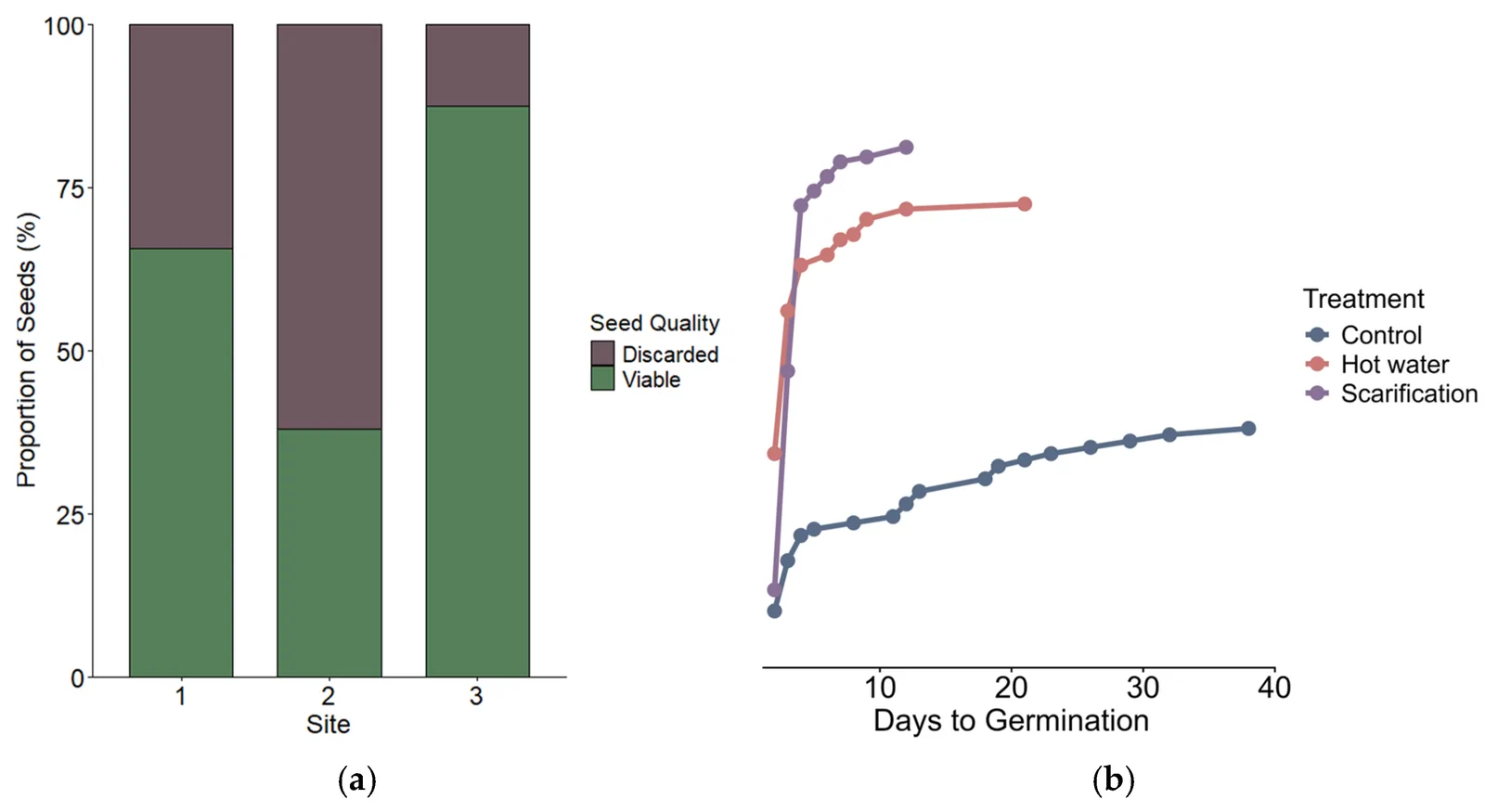
X-ray-assessed seed quality across collection sites and germination responses to dormancy-breaking treatments in minnarityi (*Acacia cyperophylla*) seeds collected from Wangkumarra Country. (**a**) Proportion of putatively viable and discarded seeds collected from the three study sites. Stacked columns show the relative contribution of each seed quality class at each site. (**b**) Cumulative germination (%) of seeds under control, hot water and scarification treatments over the 40-day monitoring period.

Seed quality was assessed for 761 seeds using X-ray imaging prior to germination testing. As no pre-flood seed cohort was available, differences in X-ray-assessed seed quality among sites cannot be attributed to flooding and are reported descriptively. Of these, 55% were classified as putatively viable based on the presence of a well-developed embryo and endosperm, while the remaining 45% were discarded due to poor seed quality (Figure 5a). Seed quality varied markedly among collection sites.

Despite producing the smallest seeds (5.6 g 1000^−1^), Site 3 had the highest proportion of putatively viable seed (87.5%), whereas Site 2 produced relatively small seeds (15.7 g 1000^−1^) with a low proportion of putatively viable seeds based on X-ray assessment. In contrast, Site 1 produced the largest seeds (47.1 g 1000^−1^ seeds) but did not have the highest seed quality.

A total of 360 seeds were included in the germination trial, of which 339 remained after 21 were removed due to fungal contamination. Of the uncontaminated seeds, 236 germinated, giving an overall germination rate of 70%.

Germination success differed markedly among treatments (Figure 5b; Table 2), with scarification producing the highest germination, followed by hot water treatment, while untreated seeds showed substantially lower germination. These results demonstrate that breaking physical dormancy greatly enhances germination in minnarityi.

Differences among treatments were also evident in the timing of germination (Figure 5b). Most seeds in the scarification and hot water treatments germinated within the first week, whereas germination in the control treatment was slower and more gradual. Accordingly, mean time to germination was substantially greater in the control treatment (9.87 ± 9.91 days) than in the hot water (3.48 ± 2.82 days) or scarification (3.64 ± 1.66 days) treatments.

Mixed-effects logistic regression showed that both dormancy-breaking treatment and seed origin significantly influenced germination success (Table 2). Relative to untreated seeds, hot water treatment increased the odds of germination 18.9-fold, while scarification increased the odds 54.3-fold. Germination probability also varied among sites, with seeds from Site 2 having significantly lower odds of germination than seeds from Site 1 (Table 2).

Germination success also varied significantly among plates (random variance for Plate = 2.64 ± 1.62; χ^2^_1_ = 32.06, *p* < 0.001), indicating that variation among experimental units contributed to germination outcomes.

## 4. Discussion

Understanding how extreme flooding affects long-lived arid-zone tree species is critical for predicting their persistence, particularly for culturally significant species, whose responses to environmental change may unfold over decades and remain undetected without long-term monitoring [33,68]. Here, we show that the culturally significant tree in Wangkumarra Country—minnarityi—was substantially affected by extreme flooding. Standing abundance declined by 30.6%, from 180 living individuals before flooding to 125 at Post6; however, the Post6 population included nine new recruits, while 116 individuals from the original pre-flood cohort survived. Losses from the pre-flood cohort exceeded recruitment, resulting in a net decline in standing abundance at the sites surveyed and a shift in demographic structure towards larger individuals with higher post-flood survival. Although recruitment occurred following flooding and viable seeds were present within populations, mortality exceeded recruitment during the six-month monitoring period. Together, these findings demonstrate that extreme flooding can alter population structure and reduce abundance in the populations monitored during this study, highlighting the importance of recruitment and early-life stage survival in population recovery.

The response of minnarityi to extreme flooding has not previously been documented, as is the case for many of Australia’s more than 1000 *Acacia* species [34,69]. This lack of demographic information limits understanding of the processes influencing reproduction, recruitment and survival [69,70,71]. Studies of *Acacia* and its close relatives (e.g., *Vachellia*, *Senegalia*) around the world consistently show that population persistence is associated with a well-represented age structure, whereas limited recruitment and under-representation of younger life stages can constrain population replacement and increase vulnerability to decline [70,72,73]. This pattern was evident in the populations monitored during this study, where low recruitment, high mortality and a post-flood population structure skewed towards mature individuals were consistent with the reduced capacity for population replacement during the monitoring period. Populations characterised by limited recruitment and ageing cohorts are commonly interpreted as being vulnerable to future decline [74,75].

Mortality was strongly size-dependent, with survival increasing substantially with plant size. Younger cohorts experienced the greatest losses, while mature trees showed higher survival following flooding. Similar demographic bottlenecks have been reported in other *Acacia* and closely related species, where seedlings and saplings are disproportionately affected by environmental disturbances, including flooding, drought, fire and grazing [34,69,75,76,77]. Because younger life stages contribute disproportionately to future population replacement, elevated mortality among these cohorts can have long-term consequences for population persistence [70,78]. Within the populations monitored during this study, the observed demographic structure highlights the importance of maintaining recruitment and younger life stages to support the replacement of adult trees.

Flood-associated sediment deposition, nutrient redistribution and changes in microsite availability can create opportunities for seedling establishment in dryland environments [79,80,81]. Although some recruitment of minnarityi occurred after flooding, mortality exceeded recruitment during the six-month monitoring period. The presence of recruits indicates that opportunities for establishment occurred following flooding. However, recruitment was assessed over only a six-month period and at three sites, and recruitment in arid-zone woody species is often highly variable through time and space. Consequently, the significance of the relatively low number of recruits observed during the study period remains uncertain and will require longer-term monitoring to evaluate.

Because minnarityi is a long-lived arid-zone tree, substantial progression among life stages would not be expected over a six-month monitoring period. The demographic dataset generated here therefore provides a valuable baseline against which future surveys can assess longer-term changes in growth, recruitment and population structure.

Changes in plant condition also support this interpretation. Condition declined rapidly following flooding and showed little evidence of recovery during the six-month monitoring period. However, pre-flood condition did not predict subsequent survival. Field observations following the flood included debris lodged within tree canopies, scour around tree bases, and localised channel erosion, indicating that plants experienced substantial flow-related disturbance. The observed losses following flooding are consistent with potential flood-related stressors, including inundation, sediment burial or physical displacement. Although multivariate soil chemistry did not differ significantly among survey periods, descriptive variation was observed in several individual soil properties. However, individual soil variables were not tested independently, and these patterns should therefore be interpreted cautiously. The ecological significance of these observed patterns remains uncertain, and longer-term monitoring will be required to determine whether they represent transient responses or longer-term changes influencing growth, reproduction and recruitment.

Despite substantial mortality and limited recruitment following flooding, minnarityi retained considerable regeneration potential through putatively viable seed reserves and high germination capacity under favourable conditions. The seed-quality assessment and germination experiments provided an important counterpoint to the demographic findings. Minnarityi exhibited strong germination potential when putatively viable seeds were available and dormancy was overcome, indicating substantial regenerative capacity under favourable conditions. This is consistent with the regeneration strategy of many arid-zone *Acacia* species and their close relatives, which maintain persistent seed reserves and rely on episodic opportunities for establishment [69,72,82]. Variation among individual seeds in dormancy and imbibition responses may also contribute to variability in germination within seed cohorts, as has been reported for other Australian *Acacia* species [52]. This potential source of variation was not specifically assessed in the present study. However, the presence of seed alone did not reliably indicate regeneration potential. Nearly half of all collected seeds were unsuitable for germination testing following X-ray assessment, and seed quality varied markedly among populations. These findings suggest that seed abundance alone is unlikely to provide a reliable indication of regeneration potential.

X-ray imaging provided a rapid means of distinguishing well-developed seeds from those that were damaged or underdeveloped, with close agreement between X-ray classifications and subsequent germination outcomes supporting its use prior to propagation or restoration activities [50]. Seed quality differed substantially among sites, with Site 3 producing the highest proportion of seeds classified as putatively viable by X-ray assessment despite having the smallest seeds, indicating that seed size was a poor predictor of X-ray-assessed seed quality. This lack of correspondence between seed size and X-ray-assessed seed quality is consistent with findings from other *Acacia* species and their close relatives indicates that seed mass alone is an unreliable indicator of regeneration potential [82,83,84].

The causes of site-level variation in seed quality remain uncertain. Prolonged inundation may have influenced seed development or parent-tree reproductive performance, but the study was not designed to isolate flood effects from other sources of variation. Differences among populations may instead reflect variation in maternal condition, reproductive investment, local environmental conditions, or underlying genetic factors such as local adaptation and founder effects [72,85,86,87]. Consequently, further research is needed to determine how environmental and genetic factors interact to influence seed development and viability in minnarityi populations.

Importantly, strong germination potential under laboratory conditions should not be interpreted as evidence of recruitment in the field. The germination experiment demonstrated that physical dormancy could be overcome under favourable conditions, but field emergence was not directly monitored. Nine new seedlings were detected at the Post3 survey, with no additional recruits detected at Post6, demonstrating that some field establishment occurred but was limited during the study period. The stage at which recruitment was most constrained could not be determined and may have involved seed germination, emergence, establishment or early seedling survival. Successful regeneration ultimately depends on progression through these stages and subsequent survival and growth [34,77]. Drought, recurrent disturbance, herbivory, grazing and livestock trampling may further constrain establishment and early survival [72,88,89]. Although direct grazing of minnarityi was not observed during surveys, the study sites occurred on an active cattle property and evidence of livestock use was widespread throughout the study area. Consequently, grazing impacts on seedling establishment and survival cannot be excluded. The germination response observed here should therefore be interpreted as evidence of germination capacity under experimental conditions rather than evidence of successful population recruitment or recovery.

Climate-driven changes in the frequency, magnitude and timing of extreme floods may further alter the balance between mortality and recruitment in arid-zone tree populations [7,90]. Although episodic flooding is a fundamental ecological process in dryland environments, increasingly variable or unpredictable flood regimes may exceed the conditions to which populations have adapted. Changes in flood magnitude, timing, duration or spatial extent may alter the balance between opportunities for regeneration and risks to established plants, particularly where extreme events exceed conditions previously experienced by populations.

Within the populations monitored in this study, repeated losses among seedlings and saplings could reduce population replacement through time, even where mature trees remain abundant and putatively viable seed is present. This possibility is particularly important given that mortality in the present study was concentrated in the youngest life stages, while progression to subsequent life stages was limited. Because changes in recruitment and juvenile survival often precede detectable declines in mature populations, the demographic consequences of extreme flooding may take years to become fully apparent [78].

A key limitation of this study is that site-specific flood depth, inundation, flow velocity and sediment deposition were not measured. Consequently, the demographic and condition changes observed represent responses following the flood event, but the relative contribution of specific flood mechanisms cannot be determined. Regional gauge records characterise the broader flood event but cannot resolve hydrological conditions at individual survey sites. Long-term demographic monitoring is therefore needed to determine whether surviving plants recover or experience delayed mortality or successfully contribute to population replacement through recruitment and progression of younger cohorts. Such monitoring will be particularly important for distinguishing short-term responses to rare flood events from longer-term shifts in population trajectories under changing climatic conditions. Continued monitoring of soil dynamics may also help clarify whether the nutrient responses observed following flooding represent transient adjustments or longer-term changes influencing growth, reproduction and recruitment [15,74,78,91]. Together, these findings suggest that the long-term persistence of minnarityi may depend on whether post-flood recruitment and survival of younger cohorts are sufficient to offset mortality associated with future extreme flood events.

## 5. Conclusions

This study provides the first quantitative assessment of the demographic structure, flood response, soil conditions, and regeneration capacity of minnarityi (*A. cyperophylla*) at three monitored populations in Wangkumarra Country following a major flood event. In the absence of previous demographic data, these findings establish a baseline for future monitoring and inform conservation planning and land management. The study highlights the importance of recruitment and seedling abundance as early indicators of population trajectories.

Although overall soil chemistry did not differ significantly among survey periods, descriptive differences were observed in several soil properties associated with nitrogen cycling, salinity and fertility. This highlights the dynamic nature of arid-zone soils in flood-affected landscapes and suggests that soil responses to flooding may be subtle, spatially variable, or emerge more slowly than demographic responses. Regeneration studies further showed that minnarityi has strong recovery potential when suitable seeds are available and physical dormancy is broken. However, marked differences in seed quality among sites indicate regeneration is shaped not only by seed availability, but also by seed quality, environmental conditions, and opportunities for seedling establishment.

The findings of this study should be interpreted within the context of the study design. Surveys were conducted on three minnarityi populations in Wangkumarra Country over a six-month period following a single flood event. The three sites occurred within the same broader drainage landscape and represent separate sampling locations rather than independent populations, limiting spatial replication and population-level inference. Minnarityi occurs across a substantially broader geographic range and occupies a variety of habitats that may differ in their exposure and response to flooding. Consequently, the demographic, soil and regeneration patterns reported here should not be assumed to represent all minnarityi populations across the species’ distribution. Longer-term monitoring across a broader range of populations and environmental settings would help to determine whether these findings are consistent across the species’ broader distribution.

As climate change continues to alter hydrological regimes across arid and semi-arid Australia, understanding the responses of culturally significant species such as minnarityi becomes increasingly important. Altered and potentially more variable extreme-event regimes, including changes in flood frequency, magnitude, timing and spatial distribution, may affect the balance between mortality and recruitment in minnarityi populations, with implications for long-term population persistence. These findings highlight the need for ongoing monitoring and adaptive management in partnership with Wangkumarra people, ensuring that ecological knowledge and cultural knowledge support the long-term stewardship of minnarityi and its associated eco-cultural values.

Based on these findings, conservation priorities for minnarityi should focus on continued demographic monitoring, particularly of seedlings and saplings, to detect shifts in recruitment and population structure; monitoring populations following major flood events to assess mortality, recovery and establishment; and conserving viable seed from multiple populations to support future restoration if required. Management should also prioritise conditions that support establishment and survival of younger life stages, which experienced the highest mortality during the monitoring period. These actions should be developed and implemented in partnership with Wangkumarra people, integrating ecological monitoring with Wangkumarra knowledge and stewardship of Wangkumarra Country.

## Figures and Tables

**Table 1:** Effects of pre-flood life stage on minnarityi (*Acacia cyperophylla*) survival six months after flooding in Wangkumarra Country. Odds ratios >1 indicate increased odds of survival relative to mature trees, whereas values <1 indicate decreased odds of survival. Mature trees had significantly greater odds of survival than seedlings and saplings.

Predictor	Odds Ratio	β	SE	z	*p*-Value
Intercept (Mature)	16.50	2.803	0.728	3.850	<0.001
Juvenile	0.23	−1.482	0.797	−1.858	0.063
Sapling	0.04	−3.122	0.776	−4.023	<0.001
Seedling	0.05	−2.998	0.813	−3.688	<0.001

**Table 2:** Effects of site and treatment on germination success. Results of mixed-effects logistic regression models examining the influence of seed origin and dormancy-breaking treatments on germination success. Site 1 and the control treatment were used as reference categories. Odds ratios greater than 1 indicate increased odds of successful germination relative to the reference category, whereas values less than 1 indicate reduced odds.

Predictor	Odds Ratio	β	SE	z	*p*-Value
Site 2 vs. Site 1	0.14	−2.00	0.66	−3.03	0.002
Site 3 vs. Site 1	0.04	−3.23	1.05	−3.08	0.002
Hot water vs. Control	18.90	2.94	0.74	3.97	<0.001
Scarification vs. Control	54.30	4.00	0.82	4.88	<0.001

## Data Availability

Data are contained within the article and Supplementary Materials. Further inquiries can be directed to the corresponding author.

## Supplementary Materials

The following supporting information can be downloaded at https://www.mdpi.com/article/10.3390/plants15213356/s1: Figure S1: Cooper Creek water volumes at Cullyamurra for 1974, 1990, 2020, 2025 floods; Table S1: PCA loadings; Table S2: Soil chemical properties measured before flooding (Pre), three months after flooding (Post3), and six months after flooding (Post6); Table S3: Numbers of seeds and Petri dishes allocated to each Site × Treatment combination; Table S4: Post-flood life-stage transitions of minnarityi individuals between the pre-flood and Post6 surveys; Analysis S1: Sensitivity analysis excluding missing individuals; Analysis S2: Life-stage transitions, recruitment and dead-or-lost calculations; Dataset S1: Demographic survey data. Dataset S2: Soil chemistry data. Dataset S3: Seed quality and germination data. Script S1: 01_Demography_and_Survival.R. Script S2: 02_Plant_Condition.R. Script S3: 03_Soil_Chemistry.R. Script S4: 04_Seed_Quality_and_Germination.R.

## Abbreviations

The following abbreviations are used in this manuscript:

Ca^2+^	Calcium Ion
CLMM	Cumulative Link Mixed Model
CSE	Culturally Significant Entities
Ctl	Control
EC	Electrical Conductivity
ECEC	Effective Cation Exchange Capacity
GLM	Generalised Linear Model
HW	Hot Water
K^+^	Potassium Ion
Mg^2+^	Magnesium Ion
MTG	Mean Time to Germination
Na^+^	Sodium Ion
NH_4_-N	Ammonium (extractable)
NO_3_-N	Nitrate (extractable)
P	Phosphorus
PCA	Principal Component Analysis
Post3	3 Months Post-Flood
Post6	6 Months Post-Flood
Pre	Pre-flood
S	Sulfur
Sc	Scarification
SE	Standard Error
TEK	Traditional Ecological Knowledge
Total C	Total Carbon
Total N	Total Nitrogen
TreeID	Unique Tree Identification Number
