# Supplementary material for: Impacts of Extreme Flooding on a Culturally Significant Tree Species: Minnarityi (Acacia cyperophylla F. Muell. ex Benth.) in Wangkumarra Country
Source: Plants (Basel). 2026 Oct 29;15(21):3356. doi: 10.3390/plants15213356 (PMC13765690; doi:10.3390/plants15213356)
Supplement: Supplementary file 1 [file plants-15-03356-s001.zip › plants-4614586-supplementary.pdf]

Supplementary Materials

Impacts of Extreme Flooding on a Culturally Significant Tree Species: Minnarityi (*Acacia cyperophylla* F. Muell. ex Benth.) in Wangkumarra Country

Mirritya Ebsworth, Caroline Jones and Rachael V. Gallagher

File Naming and Submission Order

S1_Supplementary_Material.docx

Figure S1. Cooper Creek water volumes at Cullyamurra for 1974, 1990, 2020, 2025 floods.
Table S1. PCA loadings.
Table S2. Soil chemical properties measured before flooding (Pre), three months after flooding (Post3), and six months after flooding (Post6).
Table S3. Numbers of seeds and Petri dishes allocated to each Site × Treatment combination in the germination experiment.
Table S4. Post-flood life-stage transitions of minnarityi (*Acacia cyperophylla*) individuals in Wangkumarra Country
Analysis S1. Survival analysis excluding missing/lost individuals.
Analysis S2. Life-stage transitions, recruitment and dead-or-lost calculations.

Description of Datasets

Dataset S1*:* Demographic survey data.
Dataset S2*:* Soil chemistry data.
Dataset S3*:* Seed quality and germination data.

Description of R Scripts

Script S1: 01_Demography_and_Survival.R.
Script S2: 02_Plant_Condition.R.
Script S3: 03_Soil_Chemistry.R.
Script S4: 04_Seed_Quality_and_Germination.R.

R Scripts

```
# DEMOGRAPHY AND PLANT-CONDITION DATA
# CREATE SURVIVAL DATA
# 1. LOGISTIC REGRESSION: HEIGHT VERSUS SURVIVAL
# 2. STACKED COLUMN CHART: PLANT COUNTS BY SITE AND LIFE STAGE
# STACKED COLUMN CHART: LIFE-STAGE COUNTS BY SURVEY PERIOD
# 3. SURVIVAL PERCENTAGE BY PRE-FLOOD LIFE STAGE
# 4 AND 10. BINOMIAL GLM: SURVIVAL BY PRE-FLOOD LIFE STAGE
# 5. CLMM: CONDITION BEFORE AND AFTER FLOODING
# FIGURE 3: STACKED CONDITION COUNTS
# 6. WILCOXON TEST: PRE-FLOOD CONDITION AND MORTALITY
# SOIL DATA
# 7. SOIL PCA AND PCA BIPLOT
# 8. SOIL HEAT MAP BY SURVEY PERIOD
# 9. PERMANOVA
# 10. TABLE 3: DIRECTIONAL CHANGES IN SOIL CHEMISTRY
# SEED-QUALITY DATA
# 11. STACKED SEED-QUALITY CHART
# GERMINATION DATA
# 12. CUMULATIVE GERMINATION RESULTS AND CHART
# 13. MEAN TIME TO GERMINATION
# 14. MIXED-EFFECTS LOGISTIC REGRESSION: TABLE 4
```

S1 Supplementary Material

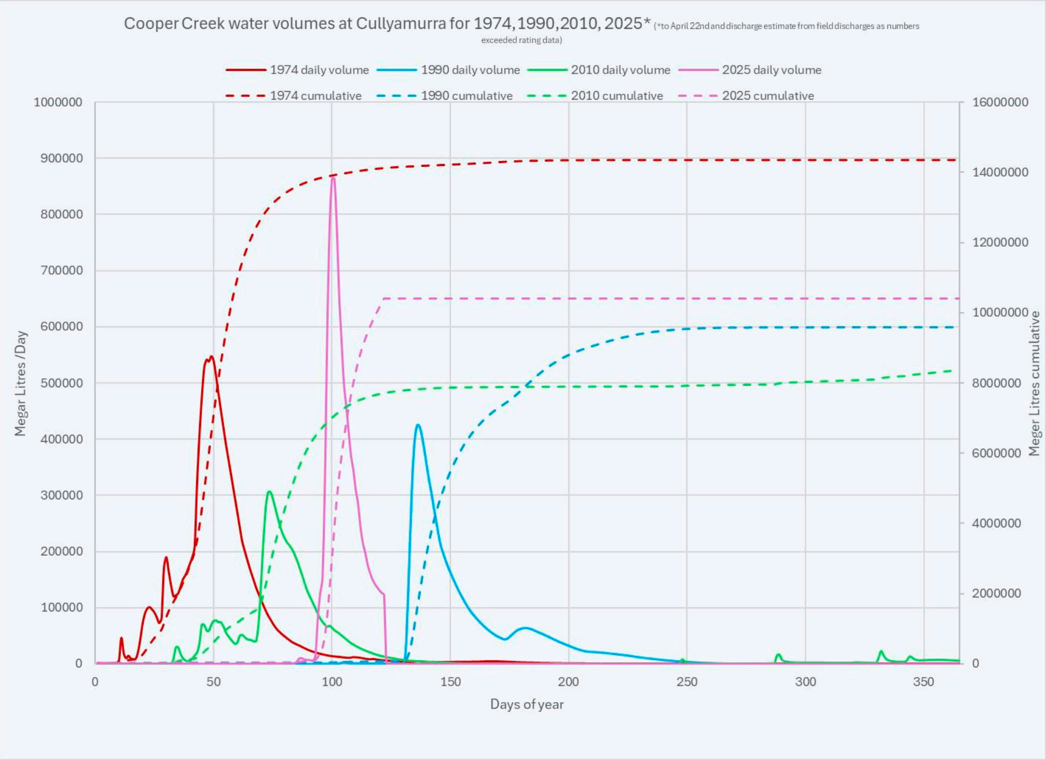

Figure S1. Cooper Creek water volumes at Cullyamurra for 1974, 1990, 2020, 2025 floods. *Image created and supplied with permission by Jimmy Stuart.*

Table S1. PCA loadings for the first two principal components.

Soil variable	PC1	PC2
pH	-0.063	0.489
EC	0.263	0.347
NO_3-N	0.148	-0.326
NH_4-N	0.221	0.379
S	0.277	0.326
P	0.327	-0.215
Total C	0.336	-0.180
Total N	0.242	-0.187
Ca	0.307	-0.162
Mg	0.292	-0.171
K	0.334	0.214
Na	0.323	0.186
ECEC	0.324	-0.117

Table S2. Soil chemical properties measured before flooding (Pre), three months after flooding (Post3), and six months after flooding (Post6). Values are mean ± SD (range), n = 3 sites per survey period

Variable	Pre	Post3	Post6
pH	7.75 ± 0.38 (7.31–8.02)	7.34 ± 0.91 (6.55–8.33)	8.07 ± 0.43 (7.58–8.39)
EC (dS m^{-1})	0.06 ± 0.04 (0.02–0.09)	0.05 ± 0.02 (0.03–0.07)	0.16 ± 0.22 (0.02–0.41)
NO_3-N (mg kg^{-1})	3.03 ± 2.70 (0.30–5.70)	8.80 ± 11.37 (0.70–21.80)	2.33 ± 1.14 (1.40–3.60)
NH_4-N (mg kg^{-1})	3.37 ± 3.10 (1.10–6.90)	1.77 ± 1.25 (0.90–3.20)	47.43 ± 75.91 (0.30–135.00)
S (mg kg^{-1})	7.07 ± 6.76 (0.50–14.00)	5.17 ± 4.04 (0.50–7.50)	13.47 ± 20.42 (0.50–37.00)
P (mg kg^{-1})	35.10 ± 26.72 (4.30–52.00)	25.00 ± 22.87 (8.00–51.00)	24.03 ± 16.04 (6.10–37.00)
Total C (%)	0.60 ± 0.46 (0.09–0.98)	0.58 ± 0.67 (0.13–1.35)	0.46 ± 0.48 (0.07–1.00)
Total N (%)	0.05 ± 0.04 (0.01–0.09)	0.12 ± 0.06 (0.07–0.18)	0.07 ± 0.05 (0.03–0.13)
Ca^{2+} ($cmol^+$ kg^{-1})	9.01 ± 7.29 (0.93–15.10)	3.81 ± 2.82 (2.10–7.06)	3.69 ± 2.66 (1.08–6.40)
Mg^{2+} ($cmol^+$ kg^{-1})	3.03 ± 3.14 (0.23–6.42)	1.32 ± 0.95 (0.58–2.40)	1.52 ± 1.09 (0.29–2.38)
K^+ ($cmol^+$ kg^{-1})	1.00 ± 0.81 (0.06–1.50)	0.31 ± 0.21 (0.13–0.54)	1.12 ± 1.34 (0.06–2.62)
Na^+ ($cmol^+$ kg^{-1})	0.33 ± 0.28 (0.03–0.59)	0.14 ± 0.11 (0.03–0.25)	0.24 ± 0.36 (0.03–0.65)
ECEC ($cmol^+$ kg^{-1})	13.33 ± 11.32 (1.20–23.60)	5.60 ± 3.91 (3.10–10.10)	6.60 ± 5.31 (1.50–12.10)

Table S3. Numbers of seeds and Petri dishes allocated to each Site × Treatment combination in the germination experiment

Site	Treatment	Seeds initially allocated	Petri dishes	Seeds included in final model
1	Control	66	11	65
1	Hot water	78	13	75
1	Scarification	78	13	74
2	Control	30	5	30
2	Hot water	36	6	31
2	Scarification	42	7	41
3	Control	6	1	6
3	Hot water	12	2	9
3	Scarification	12	2	8
**Total**		**360**	**60**	**339**

Table S4. Post-flood life-stage transitions of minnarityi (Acacia cyperophylla) individuals in Wangkumarra Country. Values represent the proportion of individuals within each pre-flood life stage that died by the six-month post-flood survey, remained in the same stage, or transitioned to a subsequent stage. Cells marked with a dash (-) represent transitions that are biologically impossible (e.g. mature trees to seedlings).

	6-months Post-flood (%)				
**Pre-flood Life Stage**	**Dead/Lost**	**Seedling**	**Sapling**	**Juvenile**	**Mature**
Seedling	54.8	35.5	9.7	0.0	0.0
Sapling	57.9	-	40.4	1.8	0.0
Juvenile	21.1	-	-	78.9	0.0
Mature	5.7	-	-	-	94.3

Analysis S1. Sensitivity analysis excluding missing individuals.

At the six-month post-flood survey, 19 of the 180 individuals recorded as alive before flooding could not be relocated. The primary analyses classified these missing/lost individuals as non-survivors. To assess the sensitivity of the results to this classification, the height- and life-stage-based survival models were repeated after excluding these 19 individuals. The relationship between pre-flood height and survival remained significant (β = 0.324 ± 0.116, z = 2.80, p = 0.005), and survival continued to differ significantly among pre-flood life stages (likelihood-ratio test: χ^2 = 21.99, df = 3, p < 0.001). Excluding missing/lost individuals increased estimated survival from 45% to 58% for seedlings and from 42% to 53% for saplings; estimates for juveniles and mature trees were unchanged because no individuals in these life stages were missing. Thus, excluding missing/lost individuals did not materially alter the conclusions of the primary survival analyses.

Analysis S2. Life-stage transition calculations.

Life-stage transitions were assessed by comparing the life stage of each individual recorded during the pre-flood survey with its life stage six months after flooding (Post6). Transition frequencies were summarised in a transition matrix, with rows representing pre-flood life stages and columns representing Post6 outcomes. Values were expressed as the proportion of individuals within each pre-flood life-stage category that were dead/lost, remained in the same category, or progressed to a subsequent category. Given the six-month monitoring period, these data are presented as a demographic baseline for future monitoring rather than as evidence of longer-term life-stage progression.

Description of Datasets

Dataset S1: Demographic survey data. Individual plant records across Pre, Post3 and Post6 surveys. Includes variables: TreeID, Site, Survey, Life stage, Observation, Condition, Height.
Dataset S2: Soil chemistry data. One record for each site × survey combination. Variables include Site, Survey and the 13 soil chemistry measurements used in PCA, PERMANOVA, z-score heat-map and percentage-change analyses.
Dataset S3: Seed viability data. Individual seed records including Site, Treatment, Plate, SeedID, Status and DaysToGerm.

Description of R Scripts

Script S1: 01_Demography_and_Survival.R. Imports and cleans demographic records; calculates abundance, mortality, recruitment and life-stage transitions; fits height-survival and life-stage survival GLMs; and generates demographic panels.
Script S2: 02_Plant_Condition.R. Prepares ordered condition classes; fits the CLMM with TreeID as a random intercept; performs the likelihood-ratio and pairwise tests; runs the Wilcoxon rank-sum test; and generates the stacked condition chart.
Script S3: 03_Soil_Chemistry.R. Prepares detection-limit values; standardised variables; performs PCA and PERMANOVA; exports PCA loadings; calculates z-scores and percentage changes; and generates the PCA biplot and heat map.
Script S4: 04_Seed_Quality_and_Germination.R. Summarises seed quality and mass; calculates cumulative germination and mean time to germination; fits the mixed-effects logistic model evaluating Site and Treatment effects on germination; and generates the seed-quality and germination figures.

R Scripts

R Packages

```
library(readxl)
library(dplyr)
library(tidyr)
library(ggplot2)
library(ggrepel)
library(scales)
library(janitor)
library(lme4)
library(lmtest)
library(ordinal)
library(emmeans)
library(nlme)
library(vegan)
```

```
############################################################
# DEMOGRAPHY AND PLANT-CONDITION DATA
############################################################

flood <- read_excel(file.choose())

flood$Time <- factor(flood$Time, levels = c("Pre", "Post3", "Post6"))
flood$Site <- factor(flood$Site, levels = c(1, 2, 3))
flood$Stage <- factor(
flood$Stage,
levels = c("Seedling", "Sapling", "Juvenile", "Mature")
)

############################################################
# CREATE SURVIVAL DATA
############################################################

pre_alive <- flood %>%
filter(
Time == "Pre",
Observation == "Alive",
Stage %in% c("Seedling", "Sapling", "Juvenile", "Mature")
) %>%
transmute(
TreeID,
Site,
Stage,
Height = `TreeHeight (m)`,
PreCondition = Condition
)

post6_status <- flood %>%
filter(Time == "Post6") %>%
select(TreeID, Observation) %>%
distinct(TreeID, .keep_all = TRUE)

survival_df <- pre_alive %>%
left_join(post6_status, by = "TreeID") %>%
mutate(
Survived = ifelse(Observation == "Alive", 1, 0),
Stage = relevel(factor(Stage), ref = "Mature")
)

############################################################
# 1. LOGISTIC REGRESSION: HEIGHT VERSUS SURVIVAL
############################################################

height_model <- glm(
Survived ~ Height,
data = survival_df,
family = binomial(link = "logit")
)

summary(height_model)
coef(summary(height_model))
exp(coef(height_model))
confint(height_model)

height_results <- data.frame(
Predictor = rownames(coef(summary(height_model))),
Beta = coef(summary(height_model))[, "Estimate"],
SE = coef(summary(height_model))[, "Std. Error"],
z = coef(summary(height_model))[, "z value"],
p_value = coef(summary(height_model))[, "Pr(>|z|)"],
Odds_Ratio = exp(coef(height_model))
)

height_results

height_graph <- ggplot(
survival_df,
aes(x = Height, y = Survived)
) +
geom_jitter(
width = 0,
height = 0.025,
size = 1.6,
alpha = 0.65,
colour = "grey20"
) +
stat_smooth(
method = "glm",
method.args = list(family = binomial(link = "logit")),
formula = y ~ x,
se = TRUE,
```

```
colour = "#536A8A",
fill = "#BFC7D5",
linewidth = 1
) +
scale_y_continuous(
limits = c(0, 1),
breaks = seq(0, 1, 0.25),
labels = percent_format(accuracy = 1)
) +
labs(
x = "Pre-flood Height (m)",
y = "Survival Probability (%)"
) +
theme_classic(base_size = 12)

height_graph

ggsave(
"Height_Survival_Logistic_Regression.tiff",
height_graph,
width = 14,
height = 14,
units = "cm",
dpi = 600,
compression = "lzw"
)

############################################################
# 2. STACKED COLUMN CHART: PLANT COUNTS BY SITE AND LIFE STAGE
############################################################

site_stage_counts <- pre_alive %>%
count(Site, Stage, name = "Count") %>%
complete(
Site,
Stage,
fill = list(Count = 0)
)

site_stage_graph <- ggplot(
site_stage_counts,
aes(x = Site, y = Count, fill = Stage)
) +
geom_col(
width = 0.72,
colour = "grey30",
linewidth = 0.25
) +
scale_fill_manual(
values = c(
"Seedling" = "#D9B446",
"Sapling" = "#86A66F",
"Juvenile" = "#5D918A",
"Mature" = "#5F7390"
),
breaks = c("Mature", "Juvenile", "Sapling", "Seedling")
) +
labs(
x = "Site",
y = "Count of Individual Plants",
fill = "Life Stage"
) +
theme_classic(base_size = 12)

site_stage_graph

ggsave(
"Life_Stage_Counts_by_Site.tiff",
site_stage_graph,
width = 15,
height = 14,
units = "cm",
dpi = 600,
compression = "lzw"
)


############################################################
# STACKED COLUMN CHART: LIFE-STAGE COUNTS BY SURVEY PERIOD
############################################################

survey_stage_counts <- flood %>%
filter(
Observation == "Alive",
Stage %in% c("Seedling", "Sapling", "Juvenile", "Mature")
```

```
) %>%
count(Time, Stage, name = "Count") %>%
complete(
Time,
Stage,
fill = list(Count = 0)
)

survey_stage_graph <- ggplot(
survey_stage_counts,
aes(x = Time, y = Count, fill = Stage)
) +
geom_col(
width = 0.72,
colour = "grey30",
linewidth = 0.25
) +
scale_fill_manual(
values = c(
"Seedling" = "#D9B446",
"Sapling" = "#86A66F",
"Juvenile" = "#5D918A",
"Mature" = "#5F7390"
),
breaks = c("Mature", "Juvenile", "Sapling", "Seedling")
) +
labs(
x = "Survey Period",
y = "Count of Individual Plants",
fill = "Life Stage"
) +
theme_classic(base_size = 12)

survey_stage_graph

ggsave(
"Life_Stage_Counts_by_Survey.tiff",
survey_stage_graph,
width = 15,
height = 14,
units = "cm",
dpi = 600,
compression = "lzw"
)

############################################################
# 3. SURVIVAL PERCENTAGE BY PRE-FLOOD LIFE STAGE
############################################################

stage_survival <- survival_df %>%
mutate(
Stage = factor(
Stage,
levels = c("Seedling", "Sapling", "Juvenile", "Mature")
)
) %>%
group_by(Stage) %>%
summarise(
Total = n(),
Survived_n = sum(Survived),
Survival_Percent = 100 * mean(Survived),
.groups = "drop"
)

stage_survival

stage_survival_graph <- ggplot(
stage_survival,
aes(x = Stage, y = Survival_Percent, fill = Stage)
) +
geom_col(
width = 0.72,
colour = "grey30",
linewidth = 0.25
) +
scale_fill_manual(
values = c(
"Seedling" = "#D9B446",
"Sapling" = "#86A66F",
"Juvenile" = "#5D918A",
"Mature" = "#5F7390"
),
guide = "none"
) +
scale_y_continuous(
```

```
limits = c(0, 100),
breaks = seq(0, 100, 25),
expand = expansion(mult = c(0, 0.02))
) +
labs(
x = "Pre-flood Life Stage",
y = "Survival (%)"
) +
theme_classic(base_size = 12)

stage_survival_graph

ggsave(
"Survival_Percent_by_Life_Stage.tiff",
stage_survival_graph,
width = 14,
height = 14,
units = "cm",
dpi = 600,
compression = "lzw"
)

############################################################
# 4 AND 10. BINOMIAL GLM: SURVIVAL BY PRE-FLOOD LIFE STAGE
############################################################

survival_df$Stage <- relevel(
factor(survival_df$Stage),
ref = "Mature"
)

stage_model <- glm(
Survived ~ Stage,
data = survival_df,
family = binomial(link = "logit")
)

stage_null <- glm(
Survived ~ 1,
data = survival_df,
family = binomial(link = "logit")
)

summary(stage_model)

stage_lrt <- anova(
stage_null,
stage_model,
test = "Chisq"
)

stage_lrt

stage_coefficients <- coef(summary(stage_model))

table1_results <- data.frame(
Predictor = rownames(stage_coefficients),
Odds_Ratio = exp(stage_coefficients[, "Estimate"]),
Beta = stage_coefficients[, "Estimate"],
SE = stage_coefficients[, "Std. Error"],
z = stage_coefficients[, "z value"],
p_value = stage_coefficients[, "Pr(>|z|)"]
)

table1_results

stage_emmeans <- emmeans(
stage_model,
~ Stage,
type = "response"
)

stage_emmeans
pairs(stage_emmeans, adjust = "tukey")

############################################################
# SENSITIVITY ANALYSIS: EXCLUDE MISSING/LOST INDIVIDUALS
############################################################

# Exclude individuals recorded as NotFound at Post6
survival_sensitivity <- survival_df %>%
filter(Observation != "NotFound") %>%
mutate(
Survived = ifelse(Observation == "Alive", 1, 0),
Stage = relevel(factor(Stage), ref = "Mature")
```

```
)

############################################################
# HEIGHT VERSUS SURVIVAL
############################################################

height_model_sensitivity <- glm(
Survived ~ Height,
data = survival_sensitivity,
family = binomial(link = "logit")
)

summary(height_model_sensitivity)
coef(summary(height_model_sensitivity))

############################################################
# SURVIVAL BY PRE-FLOOD LIFE STAGE
############################################################

stage_model_sensitivity <- glm(
Survived ~ Stage,
data = survival_sensitivity,
family = binomial(link = "logit")
)

stage_null_sensitivity <- glm(
Survived ~ 1,
data = survival_sensitivity,
family = binomial(link = "logit")
)

summary(stage_model_sensitivity)

anova(
stage_null_sensitivity,
stage_model_sensitivity,
test = "Chisq"
)

############################################################
# SURVIVAL PERCENTAGE BY LIFE STAGE
############################################################

stage_survival_sensitivity <- survival_sensitivity %>%
group_by(Stage) %>%
summarise(
Total = n(),
Survived_n = sum(Survived),
Survival_Percent = 100 * mean(Survived),
.groups = "drop"
)

stage_survival_sensitivity

############################################################
# ESTIMATED SURVIVAL AND PAIRWISE COMPARISONS
############################################################

stage_emmeans_sensitivity <- emmeans(
stage_model_sensitivity,
~ Stage,
type = "response"
)

stage_emmeans_sensitivity

pairs(
stage_emmeans_sensitivity,
adjust = "tukey"
)

############################################################
# 5. CLMM: CONDITION BEFORE AND AFTER FLOODING
############################################################

analysis_ids <- flood %>%
filter(
Time == "Pre",
Observation == "Alive"
) %>%
pull(TreeID)

condition_data <- flood %>%
filter(
TreeID %in% analysis_ids,
```

```
Time %in% c("Pre", "Post3", "Post6")
) %>%
mutate(
Condition4 = case_when(
Condition == "Good" ~ "Good",
Condition == "Fair" ~ "Fair",
Condition == "Poor" ~ "Poor",
Condition %in% c(
"Dead",
"Dead (standing)",
"Dead (fallen)",
"Dead Standing",
"Dead Fallen"
) ~ "Dead",
Observation %in% c("Dead", "NotFound") ~ "Dead",
TRUE ~ NA_character_
),
Condition4 = ordered(
Condition4,
levels = c("Dead", "Poor", "Fair", "Good")
),
Time = factor(
Time,
levels = c("Pre", "Post3", "Post6")
),
TreeID = factor(TreeID)
) %>%
filter(!is.na(Condition4))

condition_null <- clmm(
Condition4 ~ 1 + (1 | TreeID),
data = condition_data,
link = "logit",
Hess = TRUE,
nAGQ = 1
)

condition_model <- clmm(
Condition4 ~ Time + (1 | TreeID),
data = condition_data,
link = "logit",
Hess = TRUE,
nAGQ = 1
)

summary(condition_model)

condition_lrt <- anova(
condition_null,
condition_model
)

condition_lrt

condition_emmeans <- emmeans(
condition_model,
~ Time
)

condition_emmeans
pairs(condition_emmeans, adjust = "tukey")
############################################################
# FIGURE 3: STACKED CONDITION COUNTS
############################################################

condition_counts <- condition_data %>%
count(Time, Condition4, name = "Count") %>%
complete(
Time,
Condition4,
fill = list(Count = 0)
) %>%
mutate(
Condition4 = factor(
Condition4,
levels = c("Good", "Fair", "Poor", "Dead")
)
)

condition_graph <- ggplot(
condition_counts,
aes(x = Time, y = Count, fill = Condition4)
) +
geom_col(
width = 0.72,
```

```
colour = "grey30",
linewidth = 0.25
) +
scale_fill_manual(
values = c(
"Good" = "#557F59",
"Fair" = "#D3AA45",
"Poor" = "#C66746",
"Dead" = "#75636B"
),
breaks = c("Dead", "Poor", "Fair", "Good")
) +
labs(
x = "Survey Period",
y = "Count of Individual Plants",
fill = "Condition"
) +
theme_classic(base_size = 12)

condition_graph

ggsave(
"Plant_Condition_by_Survey.tiff",
condition_graph,
width = 15,
height = 14,
units = "cm",
dpi = 600,
compression = "lzw"
)

############################################################
# 6. WILCOXON TEST: PRE-FLOOD CONDITION AND MORTALITY
############################################################

condition_survival <- survival_df %>%
mutate(
PreConditionScore = case_when(
PreCondition == "Poor" ~ 1,
PreCondition == "Fair" ~ 2,
PreCondition == "Good" ~ 3,
TRUE ~ NA_real_
),
SurvivalGroup = factor(
Survived,
levels = c(0, 1),
labels = c("Died", "Survived")
)
) %>%
filter(!is.na(PreConditionScore))

table(
condition_survival$PreCondition,
condition_survival$SurvivalGroup
)

condition_wilcox <- wilcox.test(
PreConditionScore ~ SurvivalGroup,
data = condition_survival,
exact = FALSE,
correct = FALSE
)

condition_wilcox

############################################################
# SOIL DATA
############################################################

soil <- read_excel(file.choose()) %>%
clean_names()

soil$survey <- factor(
soil$survey,
levels = c("Pre", "Post3", "Post6")
)

soil$site <- factor(soil$site)

soil_variables <- c(
"p_h",
"ec",
"no3_n",
"nh4_n",
"s",
```

```
"p",
"tc",
"tn",
"ca",
"mg",
"k",
"na",
"ecec"
)

soil_labels <- c(
"p_h" = "pH",
"ec" = "EC",
"no3_n" = "NO3-N",
"nh4_n" = "NH4-N",
"s" = "S",
"p" = "P",
"tc" = "Total C",
"tn" = "Total N",
"ca" = "Ca",
"mg" = "Mg",
"k" = "K",
"na" = "Na",
"ecec" = "ECEC"
)

############################################################
# 7. SOIL PCA AND PCA BIPLOT
############################################################

soil_pca_data <- soil %>%
select(all_of(soil_variables))

soil_pca <- prcomp(
soil_pca_data,
center = TRUE,
scale. = TRUE
)

summary(soil_pca)
round(soil_pca$rotation[, 1:2], 3)

pca_scores <- as.data.frame(soil_pca$x) %>%
mutate(
Site = soil$site,
Survey = soil$survey
)

pca_loadings <- as.data.frame(soil_pca$rotation) %>%
mutate(
Variable = rownames(soil_pca$rotation),
Label = soil_labels[Variable]
)

arrow_multiplier <- 5.5

pca_loadings <- pca_loadings %>%
mutate(
PlotPC1 = PC1 * arrow_multiplier,
PlotPC2 = PC2 * arrow_multiplier
)

pca_graph <- ggplot() +
geom_hline(
yintercept = 0,
linetype = "dashed",
colour = "grey75"
) +
geom_vline(
xintercept = 0,
linetype = "dashed",
colour = "grey75"
) +
geom_point(
data = pca_scores,
aes(
x = PC1,
y = PC2,
colour = Survey
),
size = 3
) +
geom_segment(
data = pca_loadings,
aes(
```

```
x = 0,
y = 0,
xend = PlotPC1,
yend = PlotPC2
),
arrow = arrow(length = grid::unit(0.15, "cm")),
colour = "grey35",
linewidth = 0.5
) +
geom_text_repel(
data = pca_loadings,
aes(
x = PlotPC1,
y = PlotPC2,
label = Label
),
colour = "grey25",
size = 3,
max.overlaps = Inf
) +
scale_colour_manual(
values = c(
"Pre" = "#6591AA",
"Post3" = "#CC796E",
"Post6" = "#80658C"
)
) +
labs(
x = paste0(
"PC1 (",
round(100 * summary(soil_pca)$importance[2, 1], 1),
"%)"
),
y = paste0(
"PC2 (",
round(100 * summary(soil_pca)$importance[2, 2], 1),
"%)"
),
colour = "Survey Period"
) +
theme_classic(base_size = 12)

pca_graph

ggsave(
"Soil_PCA_Biplot.tiff",
pca_graph,
width = 16,
height = 14,
units = "cm",
dpi = 600,
compression = "lzw"
)

############################################################
# 8. SOIL HEAT MAP BY SURVEY PERIOD
############################################################

soil_z <- soil

soil_z[soil_variables] <- scale(
soil[soil_variables],
center = TRUE,
scale = TRUE
)

soil_heatmap_data <- soil_z %>%
select(survey, all_of(soil_variables)) %>%
pivot_longer(
cols = all_of(soil_variables),
names_to = "Variable",
values_to = "Zscore"
) %>%
group_by(survey, Variable) %>%
summarise(
Mean_Zscore = mean(Zscore, na.rm = TRUE),
.groups = "drop"
) %>%
mutate(
VariableLabel = factor(
soil_labels[Variable],
levels = rev(unname(soil_labels))
)
)
```

```
soil_heatmap <- ggplot(
soil_heatmap_data,
aes(
x = survey,
y = VariableLabel,
fill = Mean_Zscore
)
) +
geom_tile(
colour = "white",
linewidth = 0.4
) +
scale_fill_gradient2(
low = "#77A6BD",
mid = "white",
high = "#CB7775",
midpoint = 0,
name = "Z-score"
) +
labs(
x = "Survey Period",
y = "Soil Variable"
) +
theme_classic(base_size = 12)

soil_heatmap

ggsave(
"Soil_Zscore_Heatmap.tiff",
soil_heatmap,
width = 14,
height = 14,
units = "cm",
dpi = 600,
compression = "lzw"
)

############################################################
# 9. PERMANOVA
############################################################

soil_scaled <- scale(
soil[soil_variables],
center = TRUE,
scale = TRUE
)

set.seed(123)

soil_permanova <- adonis2(
soil_scaled ~ survey,
data = soil,
method = "euclidean",
permutations = 999
)

soil_permanova

soil_distance <- dist(
soil_scaled,
method = "euclidean"
)

soil_dispersion <- betadisper(
soil_distance,
soil$survey
)

anova(soil_dispersion)

set.seed(123)

permutest(
soil_dispersion,
permutations = 999
)

############################################################
# 10. TABLE 3: DIRECTIONAL CHANGES IN SOIL CHEMISTRY
############################################################

soil_means <- soil %>%
group_by(survey) %>%
summarise(
across(
```

```
all_of(soil_variables),
~ mean(.x, na.rm = TRUE)
),
.groups = "drop"
)

pre_means <- soil_means %>%
filter(survey == "Pre") %>%
select(-survey)

post3_means <- soil_means %>%
filter(survey == "Post3") %>%
select(-survey)

post6_means <- soil_means %>%
filter(survey == "Post6") %>%
select(-survey)

change_post3 <- 100 * (
as.numeric(post3_means[1, ]) -
as.numeric(pre_means[1, ])
) / as.numeric(pre_means[1, ])

change_post6 <- 100 * (
as.numeric(post6_means[1, ]) -
as.numeric(pre_means[1, ])
) / as.numeric(pre_means[1, ])

soil_change_table <- data.frame(
Variable = unname(soil_labels[soil_variables]),
Post3_Change_Percent = round(change_post3, 1),
Post6_Change_Percent = round(change_post6, 1)
)

soil_change_table

largest_increases <- soil_change_table %>%
filter(Post6_Change_Percent > 0) %>%
arrange(desc(Post6_Change_Percent))

largest_decreases <- soil_change_table %>%
filter(Post6_Change_Percent < 0) %>%
arrange(Post6_Change_Percent)

largest_increases
largest_decreases

############################################################
# SEED-QUALITY DATA
############################################################

seed_quality_raw <- read_excel(file.choose()) %>%
clean_names()

site_column <- grep(
"^site",
names(seed_quality_raw),
value = TRUE
)[1]

viable_column <- grep(
"potentially_viable",
names(seed_quality_raw),
value = TRUE
)[1]

discarded_column <- grep(
"unlikely|unlikley|discard",
names(seed_quality_raw),
value = TRUE
)[1]

seed_quality <- seed_quality_raw %>%
transmute(
Site = .data[[site_column]],
Viable = as.numeric(.data[[viable_column]]),
Discarded = as.numeric(.data[[discarded_column]])
) %>%
filter(!is.na(Site)) %>%
group_by(Site) %>%
summarise(
Viable = sum(Viable, na.rm = TRUE),
Discarded = sum(Discarded, na.rm = TRUE),
.groups = "drop"
) %>%
```

```
pivot_longer(
cols = c(Viable, Discarded),
names_to = "SeedQuality",
values_to = "Count"
) %>%
group_by(Site) %>%
mutate(
Percent = 100 * Count / sum(Count)
) %>%
ungroup() %>%
mutate(
SeedQuality = factor(
SeedQuality,
levels = c("Viable", "Discarded")
)
)

############################################################
# 11. STACKED SEED-QUALITY CHART
############################################################

seed_quality_graph <- ggplot(
seed_quality,
aes(
x = factor(Site),
y = Percent,
fill = SeedQuality
)
) +
geom_col(
width = 0.72,
colour = "grey30",
linewidth = 0.25
) +
scale_fill_manual(
values = c(
"Viable" = "#557F59",
"Discarded" = "#75636B"
),
breaks = c("Discarded", "Viable")
) +
scale_y_continuous(
limits = c(0, 100),
breaks = seq(0, 100, 25),
expand = c(0, 0)
) +
labs(
x = "Site",
y = "Proportion of Seeds (%)",
fill = "Seed Quality"
) +
theme_classic(base_size = 12)

seed_quality_graph

ggsave(
"Seed_Quality_by_Site.tiff",
seed_quality_graph,
width = 14,
height = 14,
units = "cm",
dpi = 600,
compression = "lzw"
)

############################################################
# GERMINATION DATA
############################################################

germ <- read_excel(file.choose())

germ <- germ %>%
mutate(
Site = factor(Site),
Treatment = factor(
Treatment,
levels = c("Control", "Hot water", "Scarification")
),
Plate = factor(Plate),
Status = as.numeric(Status),
DaysToGerm = as.numeric(DaysToGerm)
)

germ_clean <- germ %>%
filter(Status != 2)
```

```
############################################################
# 12. CUMULATIVE GERMINATION RESULTS AND CHART
############################################################

germination_days <- sort(
unique(
germ_clean$DaysToGerm[
germ_clean$Status == 1 &
!is.na(germ_clean$DaysToGerm)
]
)
)

cumulative_germination <- expand_grid(
Treatment = levels(germ_clean$Treatment),
Day = germination_days
) %>%
rowwise() %>%
mutate(
TotalSeeds = sum(
germ_clean$Treatment == Treatment,
na.rm = TRUE
),
CumulativeGerminated = sum(
germ_clean$Treatment == Treatment &
germ_clean$Status == 1 &
germ_clean$DaysToGerm <= Day,
na.rm = TRUE
),
CumulativePercent = 100 *
CumulativeGerminated / TotalSeeds
) %>%
ungroup() %>%
mutate(
Treatment = factor(
Treatment,
levels = c("Control", "Hot water", "Scarification")
)
)

cumulative_germination

cumulative_graph <- ggplot(
cumulative_germination,
aes(
x = Day,
y = CumulativePercent,
colour = Treatment
)
) +
geom_line(linewidth = 1) +
geom_point(size = 2.5) +
scale_colour_manual(
values = c(
"Control" = "#536A8A",
"Hot water" = "#CC796E",
"Scarification" = "#80658C"
)
) +
scale_y_continuous(
limits = c(0, 100),
breaks = seq(0, 100, 20),
expand = c(0, 0)
) +
scale_x_continuous(
limits = c(0, 40),
breaks = seq(0, 40, 10)
) +
labs(
x = "Days to Germination",
y = "Cumulative Germination (%)",
colour = "Treatment"
) +
theme_classic(base_size = 12)

cumulative_graph

ggsave(
"Cumulative_Germination_by_Treatment.tiff",
cumulative_graph,
width = 15,
height = 14,
units = "cm",
dpi = 600,
```

```
compression = "lzw"
)

################################################################
# 13. MEAN TIME TO GERMINATION
################################################################

mean_time_germination <- germ_clean %>%
filter(
Status == 1,
!is.na(DaysToGerm)
) %>%
group_by(Treatment) %>%
summarise(
N = n(),
MeanTimeToGermination = mean(DaysToGerm),
SD = sd(DaysToGerm),
SE = SD / sqrt(N),
.groups = "drop"
)

mean_time_germination

################################################################
# 14. MIXED-EFFECTS LOGISTIC REGRESSION: TABLE 4
################################################################

germ_clean$Site <- relevel(
factor(germ_clean$Site),
ref = "1"
)

germ_clean$Treatment <- relevel(
factor(germ_clean$Treatment),
ref = "Control"
)

germ_clean$Plate <- factor(germ_clean$Plate)

germination_glmm <- glmer(
Status ~ Site + Treatment + (1 | Plate),
data = germ_clean,
family = binomial(link = "logit"),
control = glmerControl(
optimizer = "bobyqa"
)
)

summary(germination_glmm)

germination_coefficients <- coef(
summary(germination_glmm)
)

table4_results <- data.frame(
Predictor = rownames(germination_coefficients),
Odds_Ratio = exp(
germination_coefficients[, "Estimate"]
),
Beta = germination_coefficients[, "Estimate"],
SE = germination_coefficients[, "Std. Error"],
z = germination_coefficients[, "z value"],
p_value = germination_coefficients[, "Pr(>|z|)"]
)

table4_results

plate_variance <- as.data.frame(
VarCorr(germination_glmm)
)

plate_variance

germination_fixed <- glm(
Status ~ Site + Treatment,
data = germ_clean,
family = binomial(link = "logit")
)

plate_lrt <- lrtest(
germination_fixed,
germination_glmm
)

plate_lrt
```
